# Small intestinal bacterial overgrowth in chronic liver disease: an updated systematic review and meta-analysis of case-control studies

**DOI:** 10.1016/j.eclinm.2024.103024

**Published:** 2024-12-31

**Authors:** Ayesha Shah, Liam Spannenburg, Parag Thite, Mark Morrison, Thomas Fairlie, Natasha Koloski, Purna C. Kashyap, Mark Pimentel, Ali Rezaie, Gregory J. Gores, Michael P. Jones, Gerald Holtmann

**Affiliations:** a Faculty of Medicine, University of Queensland, Australia; b Department of Gastroenterology & Hepatology, Princess Alexandra Hospital, Australia; c Translational Research Institute, QLD, Australia; d Faculty of Medicine, University of Queensland Frazer Institute, Woolloongabba, QLD, Australia; e Division of Gastroenterology and Hepatology, Mayo Clinic, Rochester, MN, USA; f Karsh Division of Gastroenterology and Hepatology, Department of Medicine, Medically Associated Science and Technology Program, Cedars-Sinai, Los Angeles, CA, USA; g Macquarie University, Department of Psychology, Sydney, NSW, Australia

**Keywords:** Chronic liver disease, Cirrhosis, Bacterial overgrowth, Meta-analysis, SIBO, Breath tests, NAFLD, MASLD

## Abstract

**Background:**

Small Intestinal Bacterial Overgrowth (SIBO) has been implicated in the pathophysiology of chronic liver disease (CLD). We conducted a systematic review and meta-analysis to assess and compare the prevalence of SIBO among CLD patients (with and without with complications of end stage liver disease) and healthy controls.

**Methods:**

Electronic databases were searched from inception up to July-2024 for case–control studies reporting SIBO in CLD. Prevalence rates, odds ratios (ORs), and 95% confidence intervals (CIs) of SIBO in patients with CLD and controls were calculated utilizing a random-effects model. The protocol was prospectively registered with PROSPERO (CRD42022379578).

**Findings:**

The final dataset included 34 case–control studies with 2130 CLD patients and 1222 controls. Overall, the odds for SIBO prevalence in CLD patients compared to controls was 6.7 (95% CI 4.6–9.7, p < 0.001). Although the prevalence of SIBO among patients with CLD with cirrhosis was higher at 42.9% (95% CI: 35.9–50.2) compared to 36.9% (95% CI: 27.4–47.6) in those without cirrhosis, this difference failed statistical significance. However, CLD patients with decompensated cirrhosis had a significantly higher prevalence of SIBO compared to those with compensated cirrhosis, with an OR of 2.6 (95% CI: 1.5–4.5, p < 0.001). Additionally, the prevalence of SIBO was significantly higher in CLD patients with portal hypertension (PHT) than in those without PHT, with an OR of 2.1 (95% CI: 1.4–3.1, p < 0.001). The highest prevalence of SIBO was observed in patients with spontaneous bacterial peritonitis (SBP) (57.7%, 95% CI 38.8–74.5), followed by patients with hepatic encephalopathy (41.0%, 95% CI 16.0–72.3) and patients with variceal bleed (39.5%, 95% CI 12.1–75.6).

**Interpretation:**

Overall, there is a significantly increased prevalence of SIBO in CLD patients compared to controls. The prevalence is even higher in CLD patients with PHT, especially those with SBP. This meta-analysis suggests that SIBO is associated with complications of CLD and potentially linked to the progression of CLD.

**Funding:**

10.13039/501100000925National Health and Medical Research Council, Centre for Research Excellence (APP170993).

**Research in context**

**Evidence before this study**

A preliminary meta-analysis published in 2017, which comprised 19 case–control studies, revealed an increased prevalence of Small Intestinal Bacterial Overgrowth (SIBO) among patients with Chronic Liver Disease (CLD) compared to healthy controls. Since then, several additional studies have further investigated the complex interactions between the impaired gut–liver axis and the influence of SIBO on the progression of CLD. To address this question, we conducted this updated systematic review and meta-analysis in accordance with the preferred reporting items for systematic reviews and meta-analysis statement requirements (PRISMA). Electronic databases including PUBMED, MEDLINE (OvidSP) and EMBASE were searched from initiation (1950) up to July 2024 for all studies assessing the prevalence of SIBO in patients with CLD. Grey literature was searched with Google and Google Scholar, and “Snowball” method (including pursuing references of references and electronic citation tracking to identify all the relevant articles) was employed. The literature search was conducted in collaboration with our librarian's expertise. Case-control studies that recruited unselected adults diagnosed with CLD, including fibrosis or cirrhosis, were eligible for inclusion if they reported the prevalence of SIBO diagnosed using clinically validated methods. These studies needed to compare the prevalence of SIBO between patients with CLD and healthy controls. We excluded cohort studies, manuscripts that did not present original data, those not published as full papers, and studies that failed to employ clinically validated methods for diagnosing SIBO in CLD. Individuals in the control group included ‘healthy asymptomatic controls’ as well as ‘patient controls’ including those undergoing evaluation for unexplained ‘gastrointestinal syndromes’ (e.g. anaemia, dyspepsia, pyrexia of unknown origin, diarrhoea). The quality of the case–control studies was evaluated using the Newcastle–Ottawa Scale (NOS), which assesses the selection of study groups, their comparability, and the ascertainment of the exposure of interest, with a maximum score of 9 stars. Notably, the majority of the included case–control studies (23/34, 67.6%) were classified as high quality, achieving a score of ≥6 using the NCOS.

**Added value of this study:**

This updated systematic review, and meta-analysis includes 34 peer-reviewed, published case–control studies from 18 different countries with 2130 patients with CLD and 1222 controls. This is the largest pooled analysis of case–control studies and reveals an 8-fold increased prevalence of SIBO in patients with CLD as compared to healthy controls.

SIBO prevalence is particularly higher in patients with decompensated cirrhosis and portal hypertension.

**Implications of all the available evidence**

The findings of this updated meta-analysis, suggests that SIBO potentially plays a role for the progression of CLD, rather than solely being a consequence of cirrhosis and portal hypertension.

However, the quality of evidence is low, and the results must be interpreted with caution. This arises mainly from the significant clinical heterogeneity due to varying selection criteria for cases and controls, limited sensitivity and specificity of SIBO diagnostic tests, and a small number of studies available for some subgroup analyses.

## Introduction

Small intestinal dysbiosis (defined as alterations of density, composition or metabolic functions of microbes colonising the small intestine) plays a vital role in the pathophysiology of various gastrointestinal^1^^,^^2^ and extra-intestinal disorders.^3^, ^4^, ^5^ Small intestinal bacterial overgrowth (SIBO) is one of the most widely recognized and studied clinical manifestation of gut microbial dysbiosis.^6^

Chronic liver disease (CLD) causes significant morbidity and mortality and contributes to a large economic and health burden. Small intestinal dysbiosis and SIBO, with subsequent low-grade mucosal immune activation and increased intestinal permeability, are now considered to play a critical role in the pathophysiology CLD.^7^ The altered permeability of the mucosal barrier enables increased passage of bacteria or their by-products into the bloodstream which promotes systemic inflammation and may result in bacterial infections and contributes to the progression of cirrhosis.^8^ An initial meta-analysis^5^ published in 2017 (including 19 case–control studies) found an increased prevalence of SIBO in patients with CLD compared to controls. However, since then several studies have been conducted, which have explored the interplay between the disrupted gut–liver axis and the role of SIBO in the progression of CLD and that SIBO could be an important risk factor in the pathogenesis of CLD of various aetiologies, particularly in metabolic (dysfunction)-associated steatotic liver disease (MASLD).^9^

Historically, the presence of ≥10^5^ colony-forming units per millilitre (CFU/ml) of colonic-type bacteria in the culture of jejunal aspirate has traditionally been considered the “gold standard” for diagnosing of SIBO. However, the culture-based approach is invasive, and has several methodological limitations, including a lack of universal acceptance in terms of the cut-off values for diagnosing SIBO.^10^ Thus, they have been replaced by breath tests in clinical settings. Although breath tests are simple, non-invasive, and easy to use, there is lack of consensus regarding the most appropriate substrates, the doses of substrates, the length of the test, sampling intervals, and cut-off values for diagnosing SIBO.^6^

Against this background, we conducted a systematic review and meta-analysis with a primary objective to determine and compare the prevalence of SIBO in patients with CLD and controls. Moreover, the increased number of case–control studies now available enabled our ability to explore the links between SIBO and: a) cirrhosis according to Child–Turcotte-Pugh (CTP) class; b) portal hypertension (PHT) and the major complications of PHT (including hepatic encephalopathy (HE), spontaneous bacterial peritonitis (SBP) and variceal bleeding); c) aetiology of CLD; d) the effect of diagnostic modality and proton pump inhibitor (PPI) use on the prevalence of SIBO in patients with CLD; and e) the effect of antibiotic therapy on symptom improvement in CLD patients with SIBO.

## Methods

### Protocol and registration

This systematic review and meta-analysis meets the preferred reporting items for systematic reviews and meta-analysis statement requirements (PRISMA).^11^^,^^12^ The protocol for this Systematic Review was prospectively registered with PROSPERO (CRD42022379578).

### Search strategy

**Fig. 1:**
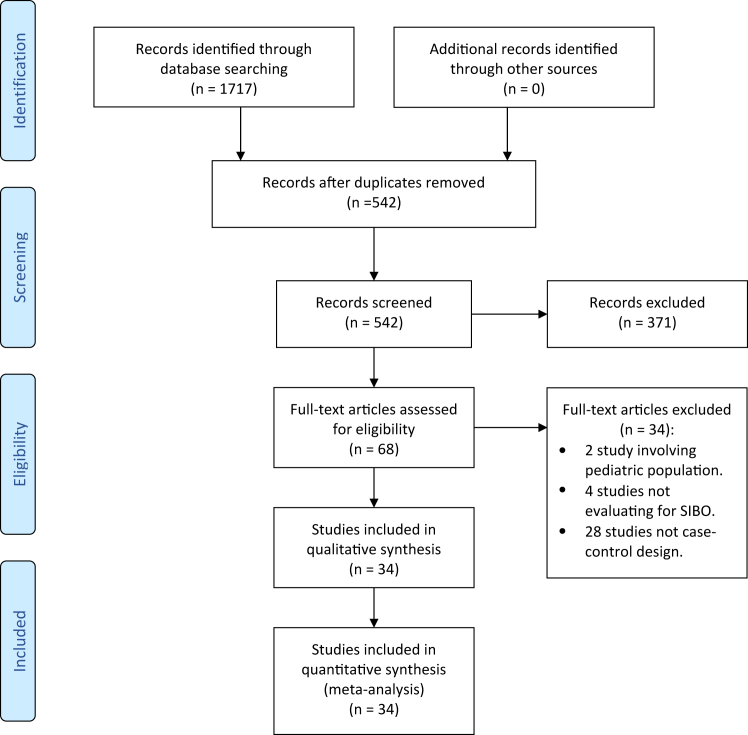
**Prisma 2009 flow diagram**.

Electronic databases, including PUBMED, MEDLINE (OvidSP) and EMBASE were searched from initiation (1950) up to July 2024 for all studies assessing all studies assessing the prevalence of SIBO in patients with CLD, including liver fibrosis and/or cirrhosis. The literature search strategy is outlined in the PRISMA flow diagram, Fig. 1 and was conducted with the assistance of our librarian. The search strategy for MEDLINE has been outlined in Figure S1 and detailed in the Supplementary File.

### Selection of studies

Two authors (L.S. and A.S.) independently screened abstracts and titles. Abstracts were eliminated if the study did not investigate the association between SIBO and CLD, (including liver fibrosis and/or cirrhosis). Full texts of the remaining articles were retrieved and reviewed.

Eligibility criteria for study inclusion are provided in Table S1 and detailed in the Supplementary File. The diagnostic criteria for SIBO in patients with CLD utilized in individual case–control studies are detailed in Table S5.

### Data extraction and quality assessment

Two authors (L.S. and P.T.) extracted data independently, and discrepancies were resolved by reference to the source publication. Data were entered into a Microsoft Excel spreadsheet ((2016 Professional edition: Microsoft Corp, Redmond, Washington, USA). The variables extracted are detailed in the Supplementary File. The quality of studies was assessed using the Newcastle–Ottawa scale (NCOS) judging the selection of the study groups, the comparability of groups and ascertainment of exposure of interest, to assign a maximum score of 9 stars.^13^

### Data analysis

In an initial step, case numbers of patients with CLD (including liver fibrosis and/or cirrhosis) and controls in the respective cohorts were determined. In a second step, the pooled odds ratio and 95% confidence intervals (CI) for the prevalence of SIBO in patients with CLD and their respective controls were calculated. Subgroup analysis and detailed in the Supplementary File. Finally, we did sensitivity analysis including only high-quality studies (assessed utilizing the NCOS), reporting the prevalence of SIBO in patients with CLD with their respective controls.

Analyses for the association between SIBO and CLD were carried out utilising the Comprehensive Meta-Analysis Software (CMA) Version 3.3.070. NJ, USA. In the results section we report the observed (unweighted) number of positive cases and total tested in addition to the weighted pooled estimates. Odds ratio and pooled prevalence estimates of disease were calculated using a random effects model^14^ to appropriately account for between-study variability. Between study variation was evaluated using Cochrane's studies^15^ and was quantified through the I^2^ index in which values close to 100 indicate substantial variation between studies while values close to zero indicate minimal between-study variation. Standard approaches (Egger Test^16^ and inspection of Funnel Plots), were applied to identify potential publication biases, for analyses when at least 10 studies included in the meta-analysis. Further, either Cochrane's test p < 0.10 or I^2^ >50% indicated substantial heterogeneity.

### Role of funding source

The funders of the study had no role in study design, data collection, data analysis, data interpretation, or writing of this manuscript.

## Results

### Selection outcome

The initial literature search revealed 1609 publications. Of these, 63 published articles appeared to be relevant for the study question and were retrieved for further evaluation. Of these, 29 were excluded, leaving 34 eligible case–control studies (Fig. 1 and Table S1). The characteristics of all the studies in the current meta-analysis are outlined in Table 1, Tables S2–S5.

**Table 1:** Characteristics of case–control studies included in the meta-analysis.

No	Author	Study year	Region	Patients with CLD n	CLD patients with cirrhosis n	Controls n	Type of control	Mode of diagnosis of SIBO	SIBO in CLD patients n (%)	SIBO in CLD patients with cirrhosis n	SIBO in controls n (%)
1	Abid et al.^17^^,^^a^	2020	Pakistan^c^	90	90	90	Healthy controls	LBT	28 (31.11)	28 (31.11)	4 (4.44)
2	Basu et al.^18^^,^^a^	2013	USA	90	45	30	Healthy controls	LBT	38 (42.22)	33 (73.3)	2 (6.66)
3	Bauer et al.^19^^,^^b^	2000	Germany	40	40	16	Healthy controls	Jejunal aspirate & culture + GBT (separate data on both tests)	29 (72.5)	29 (72.5)	2 (12.5)
4	Bjornsson et al.^20^^,^^a^	2005	Sweden	22	NA	18	Healthy controls	Jejunal aspirate and culture	1 (4.54)	NA	0 (0)
5	Bode et al.^21^^,^^b^	1993	Germany	24	NA	42	Healthy controls	LBT	8 (33.33)	NA	7 (16.66)
6	Bode et al.^22^^,^^a^	1984	Germany	27	10	13	Hospitalised patients	Jejunal aspirate and culture	13 (48.15)	4 (40)	1 (7.69)
7	Chesta et al.^23^^,^^a^	1993	Chile^c^	16	16	8	Healthy controls	LBT	7 (43.75)	7 (43.75)	0 (0)
8	Chesta et al.^24^^,^^a^	1991	Chile^c^	36	36	17	NA	Jejunal aspirate &culture + LBT (separate data on both tests)	19 (52.77)	19 (52.77)	2 (11.76)
9	Fialho et al.^25^^,^^b^	2016	USA	104	NA	268	Patients of a gastroenterology clinic	GBT	64 (61.54)	NA	77 (28.73)
10	Fitriakusumah et al.^26^^,^^b^	2019	Indonesia^c^	115	NA	45	Patients of a hepatobiliary clinic	GBT	36 (31.03)	NA	19 (42.22)
11	Ghoshal et al.^27^^,^^a^	2017	India^c^	35	NA	12	Constipation-predominant IBS patients	GBT	7 (20)	NA	0 (0)
12	Gunnarsdottir et al.^28^^,^^b^	2003	Sweden	24	24	32	Healthy controls	Jejunal aspirate and culture	4 (16.66)	4 (16.66)	0 (0)
13	Jun et al.^29^^,^^a^	2010	Korea	53	53	42	Patients of a gastroenterology clinic	LBT	32 (60.38)	32 (60.38	12 (28.57)
14	Kapil et al.^30^^,^^a^	2016	India^c^	32	NA	50	Healthy controls	Duodenal aspirate and culture	12 (37.5)	NA	0 (0)
15	Kiow et al.^31^^,^^b^	2019	Canda	52	NA	40	Healthy controls	BT (GBT/LBT unspecified)	19 (36.54)	NA	1 (2.5)
16	Lakshmi et al.^32^^,^^a^	2010	India^c^	174	174	51	Healthy controls	GBT	42 (24.14)	42 (24.14)	1 (1.96)
17	Liu et al.^33^^,^^b^	2021	China^c^	150	150	30	Healthy controls	LBT	66 (44)	66 (44)	1 (3.33)
18	Mao et al.^34^^,^^a^	2019	China^c^	46	NA	24	Healthy controls	LBT	29 (63.04)	NA	6 (25)
19	Miele et al.^35^^,^^a^	2009	Italy	35	NA	24	Healthy controls	GBT	21 (60)	NA	5 (20.83)
20	Morencos et al.^36^^,^^b^	1995	Spain^c^	89	89	40	Healthy controls	GBT	27 (30.34)	27 (35)	0 (0)
21	Nongthongbam et al.^37^^,^^b^	2015	India^c^	171	NA	16	Healthy controls	GBT	35 (20.47)	NA	0 (0)
22	Pande et al.^38^^,^^b^	2009	India^c^	53	53	13	Healthy controls	GBT	26 (49.06)	26 (49.06)	1 (7.69)
23	Sabate et al.^39^^,^^a^	2008	France	127	NA	40	Healthy controls	GBT	20 (15.75)	NA	1 (2.5)
24	Sajjad et al.^40^^,^^a^	2005	UK	12	NA	11	Healthy controls	GBT	6 (50)	NA	1 (9.09)
25	Scarpellini et al.^41^^,^^a^	2010	Italy	56	56	48	Healthy controls	GBT	29 (51.79)	29 (51.79)	2 (4.16)
26	Scarpellini et al.^42^^,^^a^	2022	Italy	52	22	14	Healthy controls	LBT	13 (25)	9 (40.9)	1 (7.14)
27	Shanab et al.^43^^,^^a^	2011	Ireland	18	NA	16	Healthy controls	LBT	14 (77.77)	NA	5 (31.25)
28	Shi et al.^44^^,^^b^	2021	China^c^	103	NA	49	Healthy controls	LBT	60 (58.25)	NA	13 (26.53)
29	Shindo et al.^45^^,^^b^	1993	Japan	27	27	17	Healthy controls	Jejunal aspirate and culture	9 (33.33)	9 (33.33)	1 (5.88)
30	Steed et al.^46^^,^^a^	2011	UK	23	23	10	Healthy controls	Duodenal biopsy and QPCR	0 (0)	0 (0)	0 (0)
31	Wigg et al.^47^^,^^b^	2001	Australia	22	NA	23	Patients of a gastroenterology clinic	LBT	11 (50)	NA	5 (21.74)
32	Xinpeng et al.^48^^,^^b^	2016	China^c^	47	47	15	Healthy controls	LBT	22 (48.81)	22 (48.81)	1 (6.66)
33	Yang et al.^49^^,^^b^	1998	Taiwan	45	45	28	Healthy controls	GBT	16 (35.55)	16 (35.55)	1 (3.57)
34	Yao et al.^50^^,^^a^	2016	China^c^	120	120	30	Healthy controls	GBT	53 (44.16)	53 (44.16)	2 (6.66)

CLD, chronic liver disease; SIBO, small intestinal bacterial overgrowth; NA, not available; LBT, lactulose breath test; GBT, glucose breath test; IBS, irritable bowel syndrome; QPCR, real time polymerase chain reaction.

a Indicates prospective.

b Indicates retrospective studies.

c Countries with a low gross domestic product (GDP) per capita (<30,000 USD/capita).

### Influence of selection criteria for controls, and risk of bias on the SIBO prevalence in CLD and controls

#### All studies

**Fig. 2:**
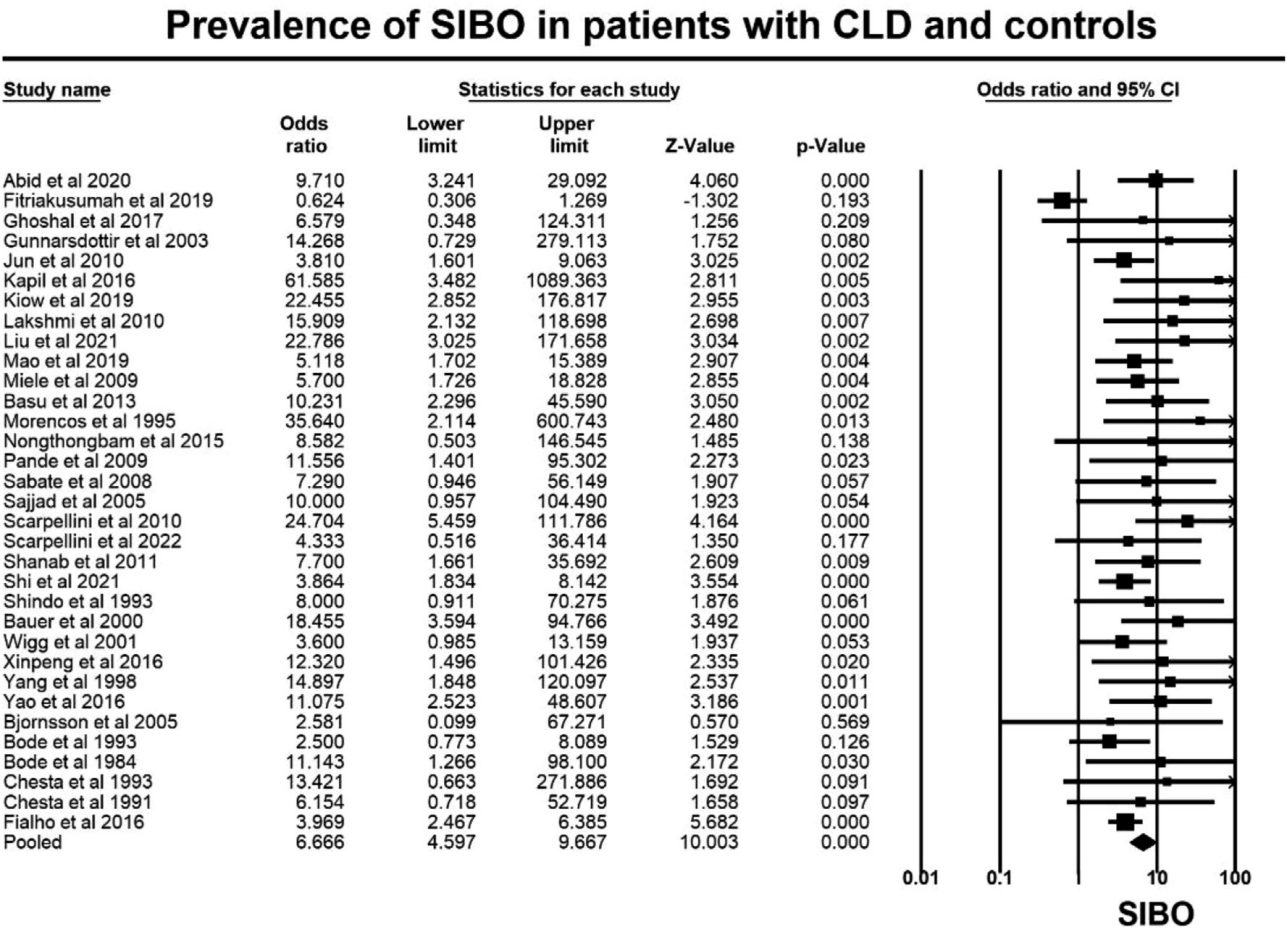
Forest plot of case–control studies showing prevalence of SIBO in patients with CLD and controls, (OR = 6.7, 95% CI 4.6–9.7, p < 0.001), (I^2^ = 48.1%, p < 0.001).

Overall, the 34 case–control studies included 2130 patients with CLD and 1222 controls. The prevalence of SIBO, in patients with CLD was 37.9% (95% CI 35.8–40.0), whereas the prevalence of SIBO in controls was 14.2% (95% CI 12.2–16.2), irrespective of the diagnostic method employed (such as quantitative culture of upper gut aspirate or glucose and lactulose hydrogen breath tests) and the criteria utilized for SIBO diagnosis Overall, the OR for SIBO in CLD as compared to controls was OR = 6.7 (95% CI 4.6–9.7, p < 0.001, Fig. 2), with moderate heterogeneity seen in the analysis (I^2^ = 48.1%, p < 0.001). Visual inspection of the funnel plot (Figure S2) showed asymmetry, suggesting the possibility of publication bias consistent with results of the Egger test (p < 0.001).

#### Impact of “control” subjects

Although most of the studies (27/34, 79.4%) utilised otherwise healthy controls, there were seven studies^22^^,^^25^, ^26^, ^27^^,^^29^^,^^46^^,^^47^ included with other patient groups deemed as controls (Table 1). When these seven studies were removed from the analysis, the OR for SIBO in patients with CLD was increased to 7.8 (95% CI 5.7–10.7, p < 0.001, Figure S3) with minimal heterogeneity in the analysis (I^2^ = 0%, p = 0.831). Visual inspection of the funnel plot (Figure S4) showed asymmetry, suggesting the possibility of publication bias consistent with results of the Egger test (p < 0.001).

### Comparison of SIBO in CLD and controls, stratified according to type of diagnostic tests

**Fig. 3:**
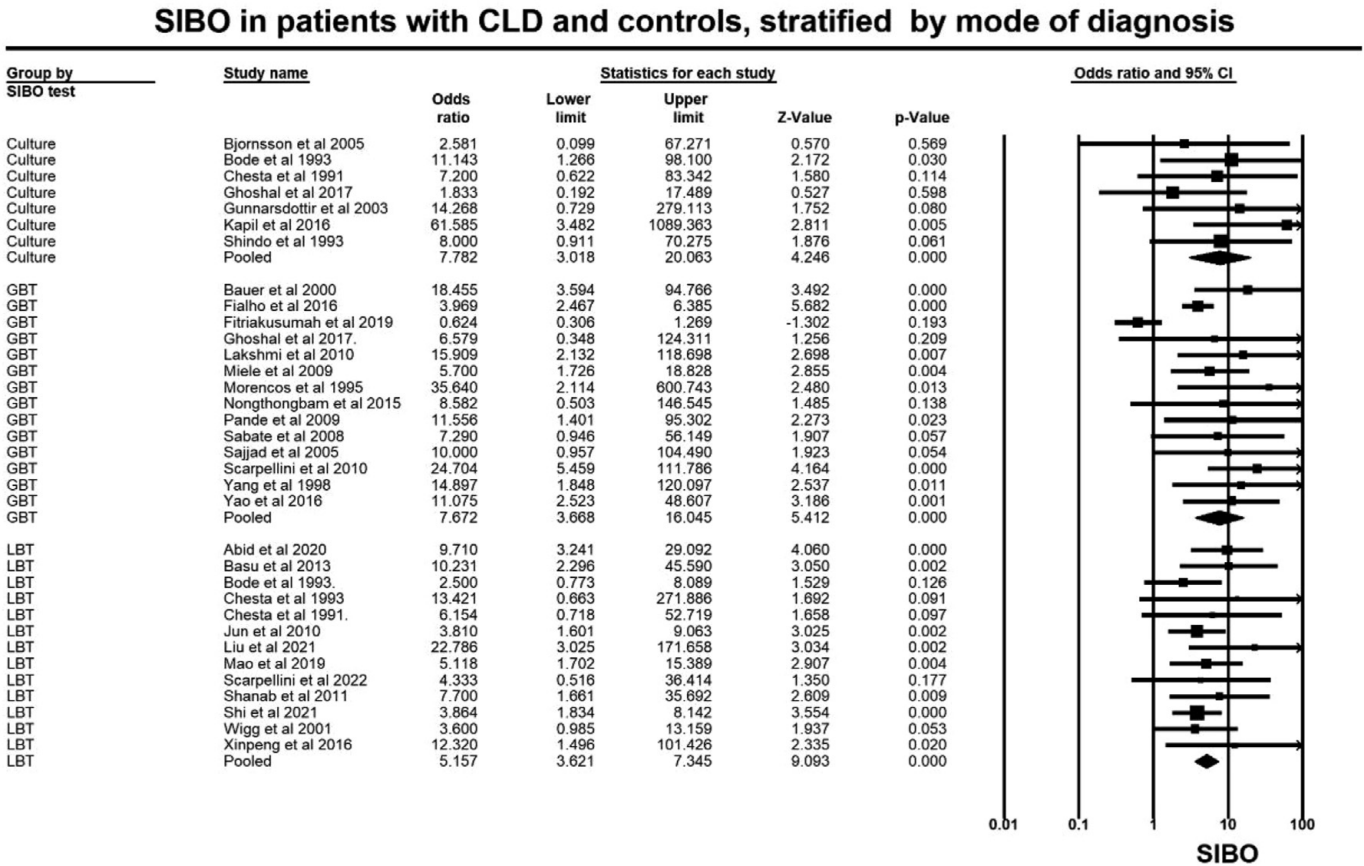
Forest plot of case–control studies showing prevalence of SIBO in patients with CLD and controls, stratified according to mode of SIBO diagnosis. Prevalence rates of SIBO in CLD patients compared to controls were highest in studies utilising small bowel culture and aspirate (OR = 7.8, 95% CI 3.0–20.1, p < 0.001), (I^2^ = 0%, p = 0.639) followed by those utilizing GBT (OR = 7.7, 95% CI 3.7–16.0, p < 0.001), (I^2^ = 71.3%, p < 0.001) and lowest in those utilising LBT (OR = 5.2, 95% CI 3.6–7.3, p < 0.001), (I^2^ = 0%, p = 0.759) for SIBO diagnosis.

As shown in Table 1, Twenty-six studies employed breath tests (thirteen using GBT, twelve using LBT, and one unspecified), five studies used small bowel aspirate/culture, two studies utilized both breath tests and small bowel aspirate/culture (with separate data on each), and one study used duodenal biopsy DNA and qPCR. Overall, the prevalence rates of SIBO in CLD patients compared to controls were highest in studies utilising small bowel aspirate/culture (OR = 7.8, 95% CI 3.0–20.1, p < 0.001) compared to studies using breath tests (OR = 5.6, 95% CI 4.0–7.6, p < 0.001). Notably, there was no heterogeneity in the studies utilizing small bowel aspirate/culture (I^2^ = 0%, p = 0.639) whereas the heterogeneity in all studies utilizing breath tests for SIBO diagnosis in CLD was large and statistically significant (I^2^ = 52.4%, p < 0.001). This could be attributed to the different substrates and the diagnostic criteria used in breath tests for SIBO diagnosis. Additionally, there were differences observed between the type of breath test used (i.e., GBT or LBT). With GBT, the OR for SIBO prevalence in patients with CLD compared to controls was greater (OR = 7.7, 95% CI 3.7–16.0, p < 0.001) than that found from studies using LBT (OR = 5.2, 95% CI 3.6–7.3, p < 0.001, Fig. 3). There was no heterogeneity in the studies utilizing LBT (I^2^ = 0%, p = 0.759) as compared to statistically significant heterogeneity in those utilizing GBT for SIBO diagnosis in CLD (I^2^ = 71.3%, p < 0.001).

### Comparison of the prevalence of SIBO in patients with CLD with a confirmed diagnosis of cirrhosis versus those without cirrhosis

Overall, there was no significant difference in the prevalence of SIBO in patients with cirrhosis (42.9%, 95% CI 35.9–50.2, 19 studies, Figure S5) compared to those without cirrhosis (36.9%, 95% CI 27.4–47.6, 18 studies, Figure S6), with substantial heterogeneity seen in both analyses, Table 2. The odds of SIBO were numerically higher in CLD patients with cirrhosis when compared to those without cirrhosis, (OR = 4.1, 95% CI 0.6–30.9, p = 0.168, 3 studies, Figure S7), with substantial heterogeneity seen in the analysis, (I^2^ = 85.0%, p < 0.001).

**Table 2:** Summary of findings.

Mode of diagnosis of SIBO in CLD	No of studies	CLD patients, n	Controls, n	SIBO in CLD patients, n	SIBO in Controls, n	Pooled prevalence rates of SIBO in CLD patients, % (95% CI)	Pooled prevalence rates of SIBO in controls, % (95% CI)	Pooled prevalence of SIBO in CLD, OR (95% CI)	Assessment of heterogeneity between studies
ALL tests (including all studies)	34	2130	1222	807	173	37.9 (35.8–40.0)	14.2 (12.2–16.2)	6.7 (4.6–9.7, p < 0.001)	I^2^ = 48.1%, p < 0.001
BREATH TESTS only	27	1923	1042	749	170	38.9 (36.7–41.1)	16.3 (14.1–18.7)	5.6 (4.0–7.6, p < 0.001)	I^2^ = 52.5%, p < 0.001
LBT	13	747	400	338	58	45.2 (41.6–48.9)	14.5 (11.2–18.3)	5.2 (3.6–7.3, p < 0.001)	I^2^ = 0%, p = 0.759
GBT	14	1176	642	411	112	34.9 (32.2–37.7)	17.4 (14.5–20.6)	7.7 (3.7–16.0, p < 0.001)	I^2^ = 71.3%, p < 0.001
CULTURE cut off 10^5^ cfu/ml	8	221	163	82	6	37.1 (30.7–43.8)	3.6 (1.3–7.8)	9.6 (4.3–21.9, p < 0.001)	I^2^ = 0%, p = 0.630
CULTURE cut off 10^3^ cfu/ml	2	62	25	41	11	66.1 (52.9–77.6)	44.0 (24.2–65.0)	9.9 (1.7–57.8, p = 0.010)	I^2^ = 0%, p = 0.650
ALL tests (including only healthy asymptomatic controls)	28	1774	819	644	59	36.3 (34.1–38.5)	7.2 (5.5–9.1)	7.8 (5.7–10.7, p < 0.001)	I^2^ = 0%, p = 0.831
All tests (including only High-quality studies)	23	1425	701	524	46	36.7 (34.2–39.3)	6.5 (4.8–8.6)	7.9 (5.6–11.2, p < 0.001)	I^2^ = 0%, p = 0.633
**SIBO in CLD with**									
Cirrhosis	19	1120	NA	455	NA	42.9 (35.9–50.2)	NA	NA	I^2^ = 79.1%, p < 0.001
Compensated cirrhosis	10	242	NA	70	NA	29.6 (18.8–43.3)	NA	NA	I^2^ = 71.4%, p < 0.001
Decompensated cirrhosis	10	437	NA	217	NA	53.5 (41.6–65.1)	NA	NA	I^2^ = 80.0%, p < 0.001
Without cirrhosis	18	1010	NA	361	NA	36.9 (27.4–47.6)	NA	NA	I^2^ = 88.4%, p < 0.001
Portal hypertension	8	219	NA	98	NA	50.0 (34.5–65.3)	NA	NA	I^2^ = 75.1%, p < 0.001
Ascites	9	282	NA	129	NA	53.7 (38.7–68.0)	NA	NA	I^2^ = 77.7%, p < 0.001
Spontaneous bacterial peritonitis	5	60	NA	34	NA	57.7 (38.8–74.5)	NA	NA	I^2^ = 32.1%, p = 0.207
Hepatic encephalopathy	4	84	NA	29	NA	41.0 (15.7–72.3)	NA	NA	I^2^ = 75.6%, p = 0.017
Variceal bleeding	2	84	NA	27	NA	39.5 (12.1–75.6)	NA	NA	I^2^ = 88.3%, p = 0.003
MASLD	14	882	NA	335	NA	43.0 (31.1–55.7)	NA	NA	I^2^ = 89.1%, p < 0.001
MASH	9	149	NA	58	NA	41.3 (26.4–58.0)	NA	NA	I^2^ = 66.5%, p = 0.002
HBV hepatitis	5	267	NA	113	NA	42.1 (34.1–50.5)	NA	NA	I^2^ = 31.2%, p = 0.213
HCV hepatitis	4	56	NA	23	NA	35.3 (10.0–73.0)	NA	NA	I^2^ = 71.6%, p = 0.014
Alcoholic liver disease	9	253	NA	86	NA	37.3 (25.7–50.5)	NA	NA	I^2^ = 66.4%, p = 0.002
Autoimmune liver disease	4	106	NA	27	NA	29.6 (21.1–39.7)	NA	NA	I^2^ = 41.0%, p = 0.165
Cryptogenic liver disease	5	83	NA	24	NA	33.3 (18.7–51.8)	NA	NA	I^2^ = 42.6%, p = 0.137
SIBO in CLD patients on PPI vs not on PPI	4	NA	NA	NA	NA	NA	NA	2.0 (0.5–7.9), p = 0.323	I^2^ = 36.9%, p = 0.191

CLD, chronic liver disease; SIBO, small intestinal bacterial overgrowth; PPI, proton pump inhibitor; MASLD, Metabolic dysfunction-associated steatotic liver disease; MASH, Metabolic dysfunction-associated steatohepatitis; NA, not applicable; n, number; OR, odds ratio; CI, confidence interval; GBT, glucose breath test; LBT, lactulose breath test; cfu/ml, colony forming units per millilitre.

### Link between SIBO and severity of cirrhosis (CTP class)

SIBO prevalence was higher in patients with decompensated cirrhosis 53.5% (95% CI 41.6–65.1, 10 studies, Figure S8) compared to those with compensated cirrhosis 29.6% (95% CI 18.8–43.3, 10 studies, Figure S9), with substantial heterogeneity seen in both analyses, Table 2. Nine studies directly compared the prevalence of SIBO in patients with both decompensated and compensated cirrhosis. The odds of SIBO prevalence in CLD patients with decompensated cirrhosis was significantly higher compared to those with compensated cirrhosis, (OR = 2.6, 95% CI 1.5–4.5, p < 0.001, Figure S10), with moderate heterogeneity seen in the analysis (I^2^ = 42.5%, p = 0.084). The prevalence of SIBO in patients with CP-A cirrhosis was lowest at 29.6% (95% CI 18.8–43.3, 10 studies), followed by CP-B cirrhosis at 43.6% (95% CI 31.7–56.3, 9 studies), and highest in patients with CP-C cirrhosis at 62.0% (95% CI 49.3–73.3, 8 studies), with substantial heterogeneity seen in these analyses (Figures not shown), Table S6.

### Link between SIBO and portal hypertension

SIBO prevalence was increased in CLD patients with PHT (50.0%, 95% CI 34.5–65.3, 8 studies), with considerable heterogeneity seen in the analysis (I^2^ = 75.1%, p < 0.001), (Figure S11). The odds of SIBO prevalence in CLD patients with PHT was significantly higher compared to those without PHT, (OR = 2.1, 95% CI 1.4–3.1, p < 0.001, Figure S12), with minimal heterogeneity seen in the analysis (I^2^ = 17.7%, p = 0.274). Furthermore, among patients with CLD who had complications of PHT, the prevalence rates of SIBO were highest in those with SBP at 57.7% (95% CI 38.8–74.5, Figure S13), followed by those with HE at 41.0% (95% CI 15.7–72.3, Figure S14), and variceal bleeding at 39.5% (95% CI 12.1–75.6, Figure S15), with substantial heterogeneity seen in these analyses, (Table 2). Finally, SIBO prevalence in CLD patients with ascites was 53.7% (95% CI 38.7–68.0), with substantial heterogeneity noted in the analysis (I^2^ = 77.7%, p < 0.001, Figure S16).

### Link between SIBO and aetiology of CLD

Next, we conducted subgroup analysis according to the aetiology of liver disease, Table S3. SIBO prevalence was highest in patients with MASLD at 43.0% (95% CI 31.1–55.7, 14 studies, Figure S17), followed by CLD due to MASH (41.3%, 95% CI 26.4–58.0, 9 studies, Figure S18), HBV (42.1%, 95% CI 34.1–50.5, 5 studies, Figure S19), HCV (35.3%, 95% CI 10.0–73.0, 4 studies, Figure S19), ALD (37.3%, 95% CI 25.7–50.5, 9 studies, Figure S20), cryptogenic liver disease (33.3%, 95% CI 18.7–51.8, 5 studies, Figure S21) and lowest in patients with autoimmune liver disease (29.6%, 95% CI 21.1–39.7, 4 studies, Figure S22). There was moderate to considerable heterogeneity in these analyses, Table 2. There was no difference in the SIBO prevalence in patients with MASLD and MASH (OR = 0.9, 95% CI 0.5–1.9, p = 0.929, 3 studies, Figure S23), with minimal heterogeneity seen in the analysis (I^2^ = 0%, p = 0.811).

### Link between SIBO and synthetic functions in patients with CLD

Seven studies reported on the synthetic function in CLD patients with and without SIBO, Table S7. SIBO positive CLD patients had moderately reduced serum albumin levels as compared to those without SIBO (standardized mean difference (SMD): −0.478, 95% CI: −1.825 to 0.869, p = 0.48, Figure S24), with substantial heterogeneity (I^2^ = 97.88%, p < 0.001). There was a small but significant increase in the coagulopathy in CLD patients with SIBO as compared to those without SIBO (SMD: 0.255, 95% CI: −0.004 to 0.514, p = 0.054, Figure S25), with minimal heterogeneity (I^2^ = 7.24%, p = 0.340). Finally, no difference in the serum bilirubin levels in CLD patients with SIBO as compared to those without SIBO (SMD: 0.035, 95% CI: −0.243 to 0.312, p = 0.807, Figure S26), with minimal heterogeneity (I^2^ = 28.18%, p = 0.243).

Results from sub-group analyses assessing SIBO prevalence in CLD patients, focusing on high-quality studies (Table S8, Figure S27 and S28), the impact of IMO in CLD patients (Table S9, Figure S29), the association between SIBO and PPI use (Table S10, Figure S30), the effectiveness of antibiotic therapy for treating SIBO in CLD (Table S11), and the relationship between SIBO and intestinal permeability, oro-cecal transit time in CLD (Table S12, Table S13) are detailed in the supplementary file.

## Discussion

This updated systematic review and meta-analysis, utilized 34 peer-reviewed, published case–control studies from 18 different countries with 2130 patients with CLD and 1222 controls. By doing so, this is not only the largest pooled analysis of case–control studies exploring the link between SIBO and patients with CLD, but also enabled additional sub-group analyses into the interplay between SIBO and CLD aetiology and assessing the role of SIBO on the progression and complications of CLD. Overall, patients with CLD had a ∼7-fold increased prevalence of SIBO compared to controls, with an odds ratio of 6.7 (95% CI 4.6–9.7, p < 0.001). SIBO prevalence rates were significantly higher in CLD patients with decompensated cirrhosis compared to those with compensated cirrhosis, (OR = 2.6, 95% CI 1.5–4.5). Additionally, subgroup analyses indicate a notable increase in SIBO prevalence among patients with CLD and PHT compared to those without PHT (OR = 2.1, 95% CI 1.4–3.1, p < 0.001), especially those with spontaneous bacterial peritonitis (57.7%, 95% CI 38.8–74.5), suggesting a potential role for SIBO in the progression and complications of CLD.

In this meta-analysis, we observed moderate to substantial heterogeneity and a high risk of publication bias across studies included in the primary and the majority of the subgroup analyses. Hence, we conducted subgroup analysis, according to the type of controls, the quality of the studies and the method of testing used for SIBO diagnosis. Firstly, we conducted subgroup analyses examining case–control studies with ‘healthy controls’; as well as conducting a sensitivity analysis restricting to those studies with ‘high-quality’ NCOS-assessment scores (i.e., with a relatively low risk of bias). The sensitivity analysis, which included only high-quality studies, revealed that the odds of SIBO in patients with CLD increased to 7.9 (95% CI 5.6–11.2, p < 0.001) compared to the primary analysis, with minimal to no heterogeneity. By including only studies that utilized culture methods for SIBO diagnosis, we found significantly higher odds of SIBO in patients with CLD compared to controls. This approach also significantly reduced heterogeneity compared to including studies that used breath testing methods in the analyses. Based on these findings we believe that the primary factor contributing to the observed heterogeneity in the analyses appears to be related to the variability in the diagnostic criteria for SIBO. Another contributing factor is the lack of uniform selection criteria for both cases and controls, including the inclusion of 'inappropriate' control subjects in some studies. Thus, we believe the wide variance in the reported SIBO prevalence rates in patients with CLD and controls relates to these two factors.

This meta-analysis also found a higher SIBO prevalence in patients with cirrhosis compared to patients without cirrhosis (OR = 4.1, 95% CI 0.6–30.9, p = 0.168), albeit not reaching statistical significance. Furthermore, we also found that SIBO was more prevalent in CLD patients with decompensated cirrhosis compared to those with compensated cirrhosis, (OR = 2.6, 95% CI 1.5–4.5, p < 0.001). These findings are consistent with the trend of SIBO prevalence being the lowest in patients with CP-A cirrhosis at 29.6% (95% CI 18.8–43.3), increasing in CP-B cirrhosis, and almost doubling in patients with CP-C cirrhosis at 62.0% (95% CI 49.3–73.3). Moreover, SIBO prevalence was increased in CLD patients with PHT at 50.0% (95% CI 34.5–65.3), with the highest rates seen in those with SBP at 57.7% (95% CI 38.8–74.5), followed by HE at 41.0% (95% CI 15.7–72.3), and variceal bleeding at 39.5% (95% CI 12.1–75.6). Limited studies examined the impact of SIBO on synthetic function in patients with CLD. Our meta-analysis found that CLD patients with SIBO showed a small but significantly increased coagulopathy and lower albumin levels than those without SIBO. Interestingly, SIBO did not appear to affect serum bilirubin levels in CLD patients. This likely suggests that SIBO is not a consequence of CLD, but SIBO and intestinal dysbiosis play a central role in the progression and complications of CLD. Recent studies^51^^,^^52^ have shown that duodenal mucosa associated microbiota in less diverse in patients with CLD as compared to healthy controls. Furthermore, this lower microbial diversity in CLD patients correlated with both increased small intestinal permeability, and serum ALT, suggesting an association with gut barrier dysfunction and hepatic inflammation.^51^ Thus SIBO and small intestinal dysbiosis likely impair gut barrier function and increase bacterial translocation, which leads to upregulation of local mucosal and systemic inflammation and infections, and contributes to hepatic decompensation and multi-organ failure, seen in end-stage liver failure.^53^

There was no significant difference in the prevalence rates of SIBO in patients with CLD based on the aetiology of their liver disease. The highest rates of SIBO were observed in patients with MASLD, while the lowest rates were found in those with autoimmune liver disease. The results of this meta-analysis are consistent with the growing body of evidence from experimental and translational research that suggests a link between SIBO and MASLD. However, a subgroup analysis could not be performed to evaluate the impact of SIBO on the severity of steatosis and inflammation in the liver.

Although we were unable to extract data to conduct subgroup analysis, three studies^35^^,^^41^^,^^47^ observed increased intestinal permeability in MASLD patients with SIBO as compared to those without SIBO. Rifaximin, a broad-spectrum, non-absorbable, semisynthetic oral antibiotic alone or in combination with lactulose are strong predictor for reducing the recurrence of hepatic encephalopathy and hospital readmissions.^54^ Antibiotics, specifically Rifaximin, have been used to treat symptoms attributable to SIBO and normalization of a positive SIBO diagnostic tests.^55^ Furthermore, we report that a short course of antibiotic therapy effectively improved symptoms, was well-tolerated, and successfully eradicated SIBO.^17^^,^^23^^,^^40^ Thus, antimicrobial therapy may be an effective therapeutic option to alleviate gastrointestinal symptoms in patients with CLD, similar to the positive outcomes observed in patients with functional gastrointestinal disorders^56^ and inflammatory bowel diseas.^57^

The role of PPI on SIBO remains controversial. A recent study^58^ using qPCR as a measure of small intestinal bacterial load reported that 56 PPI users had a significantly increased duodenal bacterial load compared to 181 non-PPI users. Weitsman et al.^59^ found no significant differences in SIBO rates between individuals using PPI and those not using PPIs, as determined by duodenal aspirate and culture (≥10^3^ CFU/ml). Although limited by small sample size, we found no significant differences in SIBO prevalence rates in CLD patients on a PPI as compared to those not on a PPI (OR = 2.0, 95% CI 0.5–7.9, p = 0.323). Similarly, but also limited by the small sample size, we found that intestinal methanogen overgrowth (IMO) was numerically higher in CLD patients compared to controls (OR = 1.8, 95% CI 0.5–6.1, p = 0.348). Recent research^2^^,^^60^^,^^61^ emphasizes the significance of methane in inhibiting gastrointestinal motility and slowing gastrointestinal transit, underscoring the importance of measuring methane levels during breath testing. Furthermore, although examined in only two studies^44^^,^^47^ the oro-cecal transit time was significantly prolonged in CLD patients diagnosed with SIBO compared to those without SIBO. However, the delayed gastrointestinal transit and subsequent bacterial stasis observed in patients with CLD and PHT^62^ could be a consequence rather than a causal factor. Therefore, further exploration of the relationship between IMO and gastrointestinal transit in CLD patients is warranted.

A strength of our meta-analysis is our inclusion of a significantly larger number of case–control studies compared to previous reports,^5^^,^^9^^,^^63^, ^64^, ^65^ even though we excluded cohort studies. Although the previous meta-analyses noted an association between SIBO and CLD, there was significant clinical heterogeneity among the studies, and with each meta-analysis focusing on a singular, and different, aspect or aetiology of CLD. However, even the current meta-analysis is not without limitations. In this systematic review and meta-analysis, for conducting the literature search we utilised two databases. While we are confident that the literature search was comprehensive, and there is a likelihood of significant overlap of journals indexed between databases, further incorporation of more databases may reduce the potential of selection bias. In addition to the challenges posed by the well-established lack of a valid and universally accepted diagnostic test for SIBO, some studies included in this meta-analysis also included both 'healthy asymptomatic subjects' and 'patients with a variety of diseases' as controls.

In this meta-analysis, we excluded cohort studies due to their limited internal validity and susceptibility to bias and confounding. Importantly, this approach allowed us to conduct meaningful analyses of subsets within these studies and explore the extent of heterogeneity present. While we acknowledge that case–control designs may not be the ideal design for estimating prevalence, we believe that the potential for bias is minimal in this instance due to the independence of the case definition and the condition (SIBO) whose prevalence is being estimated. However, another limitation worth noting is the small sample size of some case–control studies, with <50 participants per arm and some sub-group analyses are based upon a small number of studies. Moreover, the links between SIBO and cirrhosis, portal hypertension, or complications of portal hypertension did not always reach statistical significance, reflecting factors such as sample size or clinical heterogeneity.

This updated meta-analysis, that includes 34 case–control studies, revealed an 8-fold higher prevalence of SIBO in patients with CLD compared to healthy controls. Moreover, the increased number of studies included in this updated meta-analysis enabled subgroup analyses, that revealed that patients with CLD and decompensated cirrhosis, especially those with portal hypertension and spontaneous bacterial peritonitis, had notably an increased prevalence of SIBO. These data suggests that SIBO likely plays a role for the progression of CLD, rather than solely being a consequence of cirrhosis and portal hypertension. The proposed interplay between small intestinal dysbiosis and subsequent low-grade small intestinal mucosal inflammation resulting in increased in intestinal epithelial permeability likely plays a role in the progression of liver disease. Furthermore, targeting SIBO with antimicrobials such as Rifaximin may aid in treating dysbiosis and potentially halt or delay the progression of liver disease. Similarly, while the PPI use seems to be a potential risk factor for SIBO in patients with CLD, these findings are constrained by the small sample size. The clinically available diagnostic tests for SIBO are widely recognized to have limited sensitivity and specificity, as well as a lack of validity and universal acceptance regarding diagnostic thresholds and criteria for defining a positive test result. Consequently, the quality of evidence is low, requiring careful interpretation of the results. It is crucial to validate these conclusions through larger, prospective, multicentre and high-quality studies, with uniformly selected control group and utilize improved diagnostic tests for SIBO. This rigorous approach will allow a more accurate assessment of the relationship between SIBO and CLD.

## Contributors

*Ayesha Shah, Liam Spannenburg and Gerald Holtmann*–study idea, concept and design, data extraction and interpretation of data, drafting of the manuscript. Ayesha Shah and Liam Spannenburg have accessed and verified the data and share equal first co-authorship.

*Parag Thite*–data extraction, drafting of the manuscript and review of final manuscript.

*Michael P Jones*–data analysis, drafting of the manuscript and review of final manuscript.

*Thomas Fairlie*–data analysis, drafting of the manuscript and review of final manuscript.

*Natasha Koloski*–drafting of the manuscript and review of final manuscript.

*Purna Kashyap*–drafting of the manuscript and review of final manuscript.

*Mark Morrison*–drafting of the manuscript and review of final manuscript.

*Mark Pimentel*–drafting of the manuscript and review of final manuscript.

*Ali Rezaie*–drafting of the manuscript and review of final manuscript.

*Gregory Gores*–drafting of the manuscript and review of final manuscript.

## Data sharing statement

Data, analytic methods, and study materials will be made available to other researchers on request.

## Declaration of interests

**GH** report to be on the advisory boards Australian Biotherapeutics, Glutagen, Bayer and received research support from Bayer, Abbott, Pfizer, Janssen, Takeda, Allergan. He serves on the Boards of the West Moreton Hospital and Health Service (WMHHS), Queensland, UQ Healthcare, Brisbane and the Gastro-Liga, Germany and is Chair of the WMHHS Board Quality and Safety Committee. He has a patent for the Brisbane aseptic biopsy device and serves as Editor of the Gastro-Liga Newsletter. He is on the Research Committee of the Royal Australasian College of Physicians. GH acknowledges funding from the National Health and Medical Research Council (NHMRC) for the Centre for Research Excellence in Digestive Health. GH holds an MRFF and an NHMRC Ideas grant.

**MM:** Patent: “Diagnostic marker for functional gastrointestinal disorders” (Australian Patent Application WO2022256861A1) via the University of Newcastle and UniQuest (University of Queensland). Research grants from Atmo Biosciences, Soho Flordis International (SFI) Australia Research, Bayer Consumer Health, and Yakult-Nature Global Grant for Gut Health; speaker's honoraria, and travel sponsorship from GenieBiome, Janssen Australia; consultancy fees from Sanofi Australia and Danone-Nutricia Australia; speaker honoraria and travel sponsorship from Perfect Company (China), and travel sponsorship from Yakult Inc (Japan). MM also acknowledges funding from NHMRC Australia, Australian Research Council, Princess Alexandra Hospital Research Foundation, Medical Research Futures Fund of Australia, Helmsley Charitable Trust via the Australasian Gastrointestinal Research foundation, and United States Department of Defense. MM serves on the science advisory board (non-remunerated) for GenieBiome, Hong Kong.

**PK:** Member of the scientific advisory board of the International Observatory of Biocodex Microbiota Institute, ad hoc advisory board member for Pendulum and Intrinsic Medicine. Chair elect of American Gastroenterology Association Center for Gut Microbiome Education and Research. Supported by funding from National Institutes of Health (NIH DK114007, DK138818).

**AR:** Has served as an advisor for Vibrant. Has served as a consultant for Ardelyx, Bausch health, Vibrant, and Alnylam. Has served on speaker bureau for Bausch health. Has received grants from Kenneth Rainin foundation. Has patent application regarding: Internal ultraviolet therapy, Breath gas analysis, measurement of hydrogen sulfide during breath testing, Compositions and methods to treat gastrointestinal diseases and disorders, Microbiome-derived gaseous sample collection system and methods, Steerable tip needle, and Endoscopic Fluid Aspiration Device. Has equity in Dieta health, GoodLFE, and Gemelli biotech.
