# Supplementary material for: Small intestinal bacterial overgrowth in chronic liver disease: an updated systematic review and meta-analysis of case-control studies
Source: eClinicalMedicine. 2024 Dec 31;80:103024. doi: 10.1016/j.eclinm.2024.103024 (PMC11751576; doi:10.1016/j.eclinm.2024.103024)
Supplement: Supplementary material [file mmc1.docx]

**Supplementary material:**

**Methods and Materials:**

- Search strategy
- Data extraction and quality assessment
- Selection of studies
- Data analysis

**Additional Results:**

- High-quality studies with low risk of bias
- Association between methane positivity on breath tests and CLD
- Effect of Proton Pump Inhibitors on the prevalence of SIBO in CLD
- Effect of antibiotic treatment on gastrointestinal symptoms in patient with CLD with SIBO
- Link between SIBO and intestinal permeability in patients with CLD
- Association between SIBO in patients with CLD and oro-cecal transit time (OCTT)

**Legend of Figures:**

**Figure S1:** Search strategy for MEDLINE**.**

**Figure S2:** Funnel plot showing prevalence SIBO in patients with CLD compared to controls.

**Figure S3:** Forest plot of case-control studies showing SIBO in patients with CLD and healthy controls, (OR=7.8, 95%CI 5.7-10.7, p<0.001), (I^2^=0, p=0.831).

**Figure S4:** Funnel plot showing prevalence SIBO in patients with CLD compared to healthy controls.

**Figure S5:** Forest plot of case-control studies showing SIBO in patients with CLD with cirrhosis, (42.9%, 95%CI 35.9-50.2), (I^2^=79.1, p<0.001).

**Figure S6:** Forest plot of case-control studies showing SIBO in patients with CLD without cirrhosis, (36.9%, 95%CI 27.4-47.6), (I^2^=88.4, p<0.001).

**Figure S7:** Forest plot of case-control studies showing SIBO in patients with CLD with cirrhosis vs without cirrhosis, (OR=4.1, 95%CI 0.6-30.9, p=0.168), (I^2^=85.0, p<0.001).

**Figure S8:** Forest plot of studies showing SIBO in patients with CLD with decompensated cirrhosis, (53.5%, 95%CI 41.6-65.1), (I^2^=80.0, p<0.001).

**Figure S9:** Forest plot of studies showing SIBO in patients with CLD with compensated cirrhosis, (29.6%, 95%CI 18.8-43.3), (I^2^=71.4, p<0.001).

**Figure S10:** Forest plot of case-control studies showing SIBO in patients with CLD with decompensated vs compensated cirrhosis, (OR=2.6, 95%CI 1.5-4.5, p<0.001), (I^2^=42.5, p=0.084).

**Figure S11:** Forest plot of case-control studies showing SIBO in patients with CLD with portal hypertension, (50.0%, 95%CI 34.5-65.3), (I^2^=75.1, p<0.001).

**Figure S12:** Forest plot of case-control studies comparing SIBO in patients with CLD with portal hypertension compared to those without portal hypertension (OR=2.1, 95%CI:1.4-3.1, p<0.001), (I^2^=17.7, p=0.274).

**Figure S13:** Forest plot of case-control studies showing SIBO in patients with CLD with spontaneous bacterial peritonitis, (57.7%, 95%CI 38.8-74.5), (I^2^=32.1, p=0.207).

**Figure S14:** Forest plot of case-control studies showing SIBO in patients with CLD with hepatic encephalopathy, (41.0%, 95%CI 15.7-72.3), (I^2^=775.6, p=0.017).

**Figure S15:** Forest plot of case-control studies showing SIBO in patients with CLD with variceal bleeding, (39.5%, 95%CI 12.1-75.6), (I^2^=88.3, p=0.003).

**Figure S16:** Forest plot of case-control studies showing SIBO in patients with CLD with ascites, (53.7%, 95%CI 38.7-68.0), (I^2^=77.7, p<0.001).

**Figure S17:** Forest plot of case-control studies showing SIBO in patients with Metabolic dysfunction-associated steatotic liver disease (MASLD), (43.0%, 95%CI 31.1-55.7), (I^2^=89.1, p<0.001).

**Figure S18:** Forest plot of case-control studies showing SIBO in patients with Metabolic dysfunction-associated steatohepatitis (MASH), (41.3%, 95%CI 26.4-58.0), (I^2^=66.5, p=0.002).

**Figure S19:** Forest plot of case-control studies showing SIBO in patients with CLD due to viral hepatitis, (41.8%, 95%CI 34.0-50.0).

**Figure S20:** Forest plot of case-control studies showing SIBO in patients with CLD due to alcoholic liver disease (ALD), (37.3%, 95%CI 25.7-50.5), (I^2^=66.4, p=0.002).

**Figure S21:** Forest plot of case-control studies showing SIBO in patients with CLD due to cryptogenic liver disease, (33.3%, 95%CI 18.7-51.8), (I^2^=42.6, p=0.137).

**Figure S22:** Forest plot of case-control studies showing SIBO in patients with CLD due to autoimmune liver disease, (29.6%, 95%CI 21.1-39.7), (I^2^=41.0, p=0.165).

**Figure S23:** Forest plot of case-control studies showing SIBO in patients with MASLD vs MASH, (OR=0.9, 95%CI 0.5-1.9, p=0.929), (I^2^=0, p=0.811).

**Figure S24:** Forest plot of case-control studies comparing serum albumin profile in SIBO positive and SIBO negative patients with CLD (SMD: -0.478, 95%CI: -1.825-0.869, p=0.487), (I^2^=97.88, p<0.001).

**Figure S25:** Forest plot of case-control studies comparing coagulation profile in SIBO positive and SIBO negative patients with CLD (SMD: 0.255, 95%CI: -0.004-0.514, p=0.054), (I^2^=7.24, p=0.340).

**Figure S26:** Forest plot of case-control studies comparing serum bilirubin in SIBO positive and SIBO negative patients with CLD (SMD: 0.035, 95%CI: -0.243-0.312, p=0.807), (I^2^=28.18, p=0.243).

**Figure S27:** Forest plot of case-control studies showing SIBO in patients with CLD and healthy controls, including only high-quality studies (OR=7.9, 95%CI 5.6-11.2, p<0.001), (I^2^=0%, p=0.633).

**Figure S28:** Funnel plot showing prevalence SIBO in patients with CLD compared to healthy controls, including only high-quality studies.

**Figure S29:** Forest plot of case-control studies showing intestinal methanogen overgrowth (IMO) in patients with CLD compared to controls, (OR=1.8, 95%CI 0.5-6.1, p=0.348), (I^2^=0, p=0.427).

**Figure S30:** Forest plot of case-control studies showing SIBO in patients with CLD on a proton pump inhibitor (PPI) vs not on a PPI (OR=2.0, 95%CI 0.5-7.9, p=0.323), (I^2^=36.9, p=0.191).

**Figure S31:** Forest plot of case-control studies comparing oro-cecal transit time (OCTT) in SIBO positive and SIBO negative patients with CLD (SMD: 0.942, 95%CI:0.570-1.314, p<0.001), (I^2^=0, p=0.783).

**Legend of Tables:**

**Table S1:** Eligibility criteria for the studies included in systematic review and meta-analysis.

**Table S2:** SIBO in patients with CLD, stratified by presence of cirrhosis, portal hypertension (PHT) and complications of PHT (spontaneous bacterial peritonitis (SBO), hepatic encephalopathy (HE) and variceal bleeding (VB)).

**Table S3:** SIBO in patients with CLD, stratified by aetiology of liver disease.

**Table S4:** Assessment of risk factors for SIBO in patients with CLD and controls in the studies included in this meta-analysis.

**Table S5:** Assessment of cut off criteria for diagnosing SIBO in patients with CLD and controls.

**Table S6:** Studies showing the prevalence of SIBO in patients with CLD with cirrhosis, stratified according to Child-Turcotte-Pugh (CTP) score.

**Table S7:** Studies assessing the effect of SIBO on synthetic functions in patients with CLD.

**Table S8:** Newcastle-Ottawa scale for assessment of quality of Case control studies included in the Systematic review and meta-analysis.

**Table S9:** Studies showing the prevalence of methane positive SIBO in patients with CLD and controls.

**Table S10:** Studies assessing the effect of PPI on SIBO prevalence in patients with CLD.

**Table S11:** Studies evaluating the effect of antibiotic treatment in patients with CLD with SIBO.

**Table S12:** Studies assessing the effect of SIBO on intestinal permeability in patients with CLD.

**Table S13:** Studies assessing the effect of SIBO on oro-cecal transit time in patients with CLD.

**Methods and Materials:**

**Search strategy**

The initial search was not limited to specific languages to capture all appropriate studies. A further advanced search was conducted. Grey literature was searched with Google and Google Scholar, and the ‘Snowball’ method was also utilised to identify all relevant articles. Case-control studies that recruited unselected adults diagnosed with CLD, including fibrosis or cirrhosis, were eligible for inclusion if they reported the prevalence of SIBO diagnosed using clinically validated methods. These studies needed to compare the prevalence of SIBO between patients with CLD and healthy controls. We excluded cohort studies, manuscripts that did not present original data, those not published as full papers, and studies that failed to employ clinically validated methods for diagnosing SIBO in CLD. Individuals in the control group included ‘healthy asymptomatic controls’ as well as ‘patient controls’ including those undergoing evaluation for unexplained ‘gastrointestinal syndromes’ (e.g. anaemia, dyspepsia, pyrexia of unknown origin, diarrhoea).

**Data extraction and quality assessment**

Data were entered into a Microsoft Excel spreadsheet ((2016 Professional edition: Microsoft Corp, Redmond, Washington, USA). The variables extracted are detailed in the supplementary file. The following information was extracted from each study independently by the two reviewers: author, year of publication, journal, study design, country, source of controls, method of diagnosis of SIBO including test duration, quantity of substrate used and the cut-off criteria for diagnosis of SIBO, mean age, sex, concurrent use of PPI and antibiotics, any significant co-morbidities including previous surgery for patients with CLD and the control group. In addition, for all patients with CLD, data regarding the mode of diagnosis of CLD (including liver fibrosis and/or cirrhosis), aetiology of CLD, CTP score, presence of portal hypertension (including complications such as HE, SBP and variceal bleeding), other clinical manifestations of CLD (hyperbilirubinemia, hypoalbuminemia, coagulopathy and malnutrition), data on intestinal permeability, treatment of SIBO in CLD patients with antibiotics and objective and subjective response post-treatment, and the prevalence of methane positivity on breath in patients with CLD and controls was recorded.

**Selection of studies**

Case-control studies, recruiting unselected adult subjects meeting diagnostic criteria for CLD, (with or without cirrhosis), that reported the prevalence of SIBO using clinically validated methods, and compared the prevalence SIBO in CLD versus controls were eligible for inclusion. If a study was interventional, both intervention and control groups were included. Studies not reporting original data, case reports or case series, animal studies, review articles, cohort or prevalence studies, those reporting on paediatric population, those not providing separate or unclear data on SIBO in CLD or those that did not use clinically validated methods to diagnose SIBO^1, 2^ were excluded. Conference abstracts that provided available data were also included in the study. Individuals in the control group included healthy controls as well as ‘patient controls’ including hospitalised patients, patients undergoing evaluation for ‘gastrointestinal syndromes’ (e.g. IBS) and patients visiting hepatobiliary and gastroenterology outpatient clinics. Disagreements between reviewers were resolved by mutual consensus after reference to the original published paper.

**Data Analysis**

Subgroup analysis stratified by diagnostic modalities, aetiology of CLD, cirrhosis according to CTP class, complications of portal hypertension (namely HE, SBP and variceal bleeding), GDP, effect of PPI, methane positivity on breath in CLD patients and controls were done. Lastly, we calculated the efficacy of antibiotic treatment in SIBO positive CLD patients. We only summarized PPIs/antibiotics data that were reported in included case-control studies.

Analyses for the association between SIBO and CLD were carried out utilising the Comprehensive Meta-Analysis Software (CMA) Version 3.3.070. NJ, USA. In the results section we report the observed (unweighted) number of positive cases and total tested in addition to the weighted pooled estimates. Odds ratio and pooled prevalence estimates of disease were calculated using a random effects model^3^ to appropriately account for between-study variability. The statistical package CMA utilized a logit transformation of proportions and the variance of the logit to estimate pooled event rates within groups and to compare event rates between groups. If one or more cells had a value of 0, then the CMA software automatically adds a fixed value of 0.5 to the respective cell for computation of log odds ratio and variance.

**Additional Results:**

***High-quality studies with low risk of bias:***

The majority (23/34, 67.6%) of the case-control studies were deemed to be of high quality, defined as a score of ≥6 using the Newcastle-Ottawa Assessment Scale (NCOS, Table S4)^4-32^, and when only these studies were used the OR for SIBO in CLD patients as compared to healthy controls was again increased, to 7.9 (95%CI 5.6-11.2, p<0.001, Figure S27) and with minimal heterogeneity (I^2^=0%, p=0.633). Visual inspection of the funnel plot (Figure S28) showed asymmetry, suggesting the possibility of publication bias consistent with the results of the Egger test (p=0.005).

**Association between methane positivity on breath tests and CLD**

Three studies^5, 17, 33^ assessed for intestinal methanogen overgrowth (IMO) in patients with CLD and controls, Table S6. Although, the prevalence of IMO in patients with CLD was higher compared to controls (OR=1.8, 95%CI 0.5-6.1, p=0.348, figure S29), this failed statistical significance with minimal heterogeneity in the analysis (I^2^=0, p=0.427).

**Effect of Proton Pump Inhibitors on the prevalence of SIBO in CLD**

Overall, four studies^5, 17, 29, 33^ looked at the association between PPI use and SIBO in patients with CLD, (Table S7). Overall, there was no significant difference in the rates of SIBO prevalence in patients with CLD on a PPI as compared to those not on a PPI, OR=2.0 (95%CI 0.5-7.9, p=0.323, Figure S30), with moderate heterogeneity seen in the analysis (I^2^=36.9, p=0.191).

**Effect of antibiotic treatment on gastrointestinal symptoms in patient with CLD with SIBO**

Three studies, which comprised 41 patients with CLD and SIBO, reported on response to antibiotic treatment, and one study each utilised rifaximin^4^, ciprofloxacin^23^ or tetracycline^10^ (Table S8). Two of these studies confirmed eradication of SIBO, through normalisation of either the LBT^4^ or GBT^23^ in 97% of patients (95%CI 84.7-99.9). However, significant improvement in gastrointestinal symptoms was reported in only one study, which used rifaximin^4^ and with a 94.1% improvement rate (95%CI 71.3-99.8).

**Link between SIBO and intestinal permeability in patients with CLD**

Four studies^6, 19, 24, 29^ assessed the effect of SIBO on intestinal permeability in patients with CLD, Table S10. However, due to heterogeneity in the methodology used to measure intestinal permeability, data could not be extracted to conduct subgroup analysis. Three studies^19, 24, 29^ observed increased intestinal permeability in MASLD patients with SIBO as compared to those without SIBO.

**Association between SIBO in patients with CLD and oro-cecal transit time (OCTT)**

Only two studies^26, 29^ examined the relationship between SIBO and OCTT in patients with CLD, and both utilized LBT for its measurement (Table S11). The OCTT was significantly longer in patients with CLD who had SIBO compared to those without SIBO, (SMD: 0.942, 95%CI:0.570-1.314, p<0.001, Figure S31) and with minimal heterogeneity (I^2^=0, p=0.783).

**Figures:**

| **Database search strategy MEDLINE(PubMed)** |
| --- |
| #1. "small bowel"[tiab:~2] OR "small intestine"[tiab:~2] OR "small intestinal"[tiab:~2] OR intestine, small[mh]   #2. "bacterial overgrowth"[tiab:~2] OR "bacteria overgrowth"[tiab:~2] OR "dysbiosis"[tiab] OR microbiome[tiab] OR  microbiota[tiab] OR flora[tiab] OR probiotic*[tiab] OR prebiotic*[tiab] OR symbiotic*[tiab] OR "breath tests"[tiab:~2] OR "breath test"[tiab:~2] OR "SIBO"[tiab] OR gastrointestinal microbiome[mh] OR breath tests[mh]   #3. liver diseases[mh] OR "liver disease"[tiab:~2] OR "liver diseases"[tiab:~2] OR "NAFLD"[tiab] OR "NASH"[tiab] OR "MASLD"[tiab] OR "MASH"[tiab] OR hepatitis[tiab] OR "Budd-Chiari syndrome"[tiab] OR cirrhosis[tiab] OR cholangitis[tiab] OR  proton pump inhibitors[mh] OR omeprazole [mh] OR "spontaneous bacterial peritonitis" OR "hepatocellular insufficiency" OR "jaundice" OR "portal hypertension" OR "proton pump inhibitors" OR "hepatic encephalopathy" OR "ascites" OR "variceal bleeding"    #4. #1 AND #2 AND #3    #5. animals[mh] NOT humans[mh]    #6. #4 NOT #5  **Limit:** 01/01/1950 **-** 07/2024  **Filters applied:** MEDLINE  **PubMed:** 542  *Article title ti, abstract ab, topic MeSH Terms, main topic MeSH Major Topic* |

**Figure S1:** Search strategy for MEDLINE**.**

**Figure S2:** Funnel plot showing prevalence SIBO in patients with CLD compared to controls.

**Figure S3:** Forest plot of case-control studies showing SIBO in patients with CLD and healthy controls, (OR=7.8, 95%CI 5.7-10.7, p<0.001), (I^2^=0, p=0.831).

**Figure S4:** Funnel plot showing prevalence SIBO in patients with CLD compared to healthy controls.

**Figure S5:** Forest plot of case-control studies showing SIBO in patients with CLD with cirrhosis, (42.9%, 95%CI 35.9-50.2), (I^2^=79.1, p<0.001).

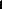


**Figure S6:** Forest plot of studies showing SIBO in patients with CLD without cirrhosis, (36.9%, 95%CI 27.4-47.6), (I^2^=88.4, p<0.001).

**Figure S7:** Forest plot of case-control studies showing SIBO in patients with CLD with cirrhosis vs without cirrhosis, (OR=4.1, 95%CI 0.6-30.9, p=0.168), (I^2^=85.0, p<0.001).

**Figure S8:** Forest plot of studies showing SIBO in patients with CLD with decompensated cirrhosis, (53.5%, 95%CI 41.6-65.1), (I^2^=80.0, p<0.001).

**Figure S9:** Forest plot of studies showing SIBO in patients with CLD with compensated cirrhosis, (29.6%, 95%CI 18.8-43.3), (I^2^=71.4, p<0.001).

**Figure S10:** Forest plot of case-control studies showing SIBO in patients with CLD with decompensated vs compensated cirrhosis, (OR=2.6, 95%CI 1.5-4.5, p<0.001), (I^2^=42.5, p=0.084).

**Figure S11:** Forest plot of case-control studies showing SIBO in patients with CLD with portal hypertension, (50.0%, 95%CI 34.5-65.3), (I^2^=75.1, p<0.001).

**Figure S12:** Forest plot of case-control studies comparing SIBO in patients with CLD with portal hypertension compared to those without portal hypertension (OR=2.1, 95%CI:1.4-3.1, p<0.001), (I^2^=17.7, p=0.274).

**Figure S13:** Forest plot of case-control studies showing SIBO in patients with CLD with spontaneous bacterial peritonitis, (57.7%, 95%CI 38.8-74.5), (I^2^=32.1, p=0.207).

**Figure S14:** Forest plot of case-control studies showing SIBO in patients with CLD with hepatic encephalopathy (HE), (41.0%, 95%CI 15.7-72.3), (I^2^=75.6, p=0.017).

**Figure S15:** Forest plot of case-control studies showing SIBO in patients with CLD with variceal bleeding, (39.5%, 95%CI 12.1-75.6), (I^2^=88.3, p=0.003).

**SIBO in CLD patients with ascites**

**Figure S16:** Forest plot of case-control studies showing SIBO in patients with CLD with ascites, (53.7%, 95%CI 38.7-68.0), (I^2^=77.7, p<0.001).

**Prevalence of SIBO in patients with MASLD**

**Figure S17:** Forest plot of case-control studies showing SIBO in patients with Metabolic dysfunction-associated steatotic liver disease (MASLD), (43.0%, 95%CI 31.1-55.7), (I^2^=89.1, p<0.001).

**Prevalence of SIBO in patients with MASH**

**Figure S18:** Forest plot of case-control studies showing SIBO in patients with Metabolic dysfunction-associated steatohepatitis (MASH), (41.3%, 95%CI 26.4-58.0), (I^2^=66.5, p=0.002).

**Figure S19:** Forest plot of case-control studies showing SIBO in patients with CLD due to viral hepatitis, (41.8%, 95%CI 34.0-50.0).

**Figure S20:** Forest plot of case-control studies showing SIBO in patients with CLD due to alcoholic liver disease (ALD), (37.3%, 95%CI 25.7-50.5), (I^2^=66.4, p=0.002).

**Figure S21:** Forest plot of case-control studies showing SIBO in patients with CLD due to cryptogenic liver disease, (33.3%, 95%CI 18.7-51.8), (I^2^=42.6, p=0.137).

**Prevalence of SIBO in patients with autoimmune liver disease**

**Figure S22:** Forest plot of case-control studies showing SIBO in patients with CLD due to autoimmune liver disease, (29.6%, 95%CI 21.1-39.7), (I^2^=41.0, p=0.165).

**Prevalence of SIBO in patients with MASLD as compared to MASH**

**Figure S23:** Forest plot of case-control studies showing SIBO in patients with Metabolic dysfunction-associated steatotic liver disease (MASLD) vs Metabolic dysfunction-associated steatohepatitis (MASH), (OR=0.9, 95%CI 0.5-1.9, p=0.929), (I^2^=0, p=0.811).

**Figure S24:** Forest plot of case-control studies comparing serum albumin profile in SIBO positive and SIBO negative patients with CLD (SMD: -0.478, 95%CI: -1.825-0.869, p=0.487), (I^2^=97.88, p<0.001).

**Figure S25:** Forest plot of case-control studies comparing coagulation profile in SIBO positive and SIBO negative patients with CLD (SMD: 0.255, 95%CI: -0.004-0.514, p=0.054), (I^2^=7.24, p=0.340).

**Figure S26:** Forest plot of case-control studies comparing serum bilirubin in SIBO positive and SIBO negative patients with CLD (SMD: 0.035, 95%CI: -0.243-0.312, p=0.807), (I^2^=28.18, p=0.243).

**Figure S27:** Forest plot of case-control studies showing SIBO in patients with CLD and healthy controls, including only high-quality studies (OR=7.9, 95%CI 5.6-11.2, p<0.001), (I^2^=0%, p=0.633).

**Figure S28:** Funnel plot showing prevalence SIBO in patients with CLD compared to healthy controls, including only high-quality studies.

**Figure S29:** Forest plot of case-control studies showing intestinal methanogen overgrowth (IMO) in patients with CLD compared to controls, (OR=1.8, 95%CI 0.5-6.1, p=0.348), (I^2^=0, p=0.427).

**Figure S30:** Forest plot of case-control studies showing SIBO in patients with CLD on a proton pump inhibitor (PPI) vs not on a PPI (OR=2.0, 95%CI 0.5-7.9, p=0.323), (I^2^=36.9, p=0.191).

**Figure S31:** Forest plot of case-control studies comparing oro-cecal transit time (OCTT) in SIBO positive and SIBO negative patients with CLD (SMD: 0.942, 95%CI:0.570-1.314, p<0.001), (I^2^=0, p=0.783).

**Table S1:** Eligibility criteria for the studies included in systematic review and meta-analysis.

| **Eligibility criteria** |
| --- |
| - Case-control studies published as full papers in peer reviewed journals or conference abstracts. |
| - Adults with a diagnosis of chronic liver disease, including liver fibrosis and cirrhosis. - Control group, referred to as ‘controls’ included ‘healthy asymptomatic controls’ as well as ‘patient controls’ including patients undergoing evaluation for unexplained gastrointestinal ‘syndromes’ (e.g., anemia, dysphagia, Barrett’s esophagus, diarrhea etc.). |
| - Clinically validated methods to diagnose small intestinal bacterial overgrowth. ** |
| - Participants not specially selected. |
| - ** Lactulose breath test, glucose breath test, or small bowel aspirate and culture (or any combination of these)^1, 34^. |

**Table S2:** SIBO in patients with CLD, stratified by presence of cirrhosis, portal hypertension (PHT) and complications of PHT (spontaneous bacterial peritonitis (SBO), hepatic encephalopathy (HE) and variceal bleeding (VB)).

| No | Author | CLD patients with cirrhosis, n | CLD patients with cirrhosis, n | | CLD patients with cirrhosis with PHT n | CLD patients with cirrhosis with PHT n | | | SIBO in CLD patients with cirrhosis n | SIBO in CLD patients with cirrhosis with PHT n | SIBO in CLD patients with cirrhosis n | | SIBO in CLD patients with cirrhosis with PHT n | | |
| --- | --- | --- | --- | --- | --- | --- | --- | --- | --- | --- | --- | --- | --- | --- | --- |
|  |  |  | Compensated cirrhosis | Decompensated cirrhosis |  | SBP | HE | VB |  |  | Compensated cirrhosis | Decompensated cirrhosis | SBP | HE | VB |
| 1 | Abid et al.^4^ | 90 | NA | NA | NA | NA | 55 |  | 28 | NA | NA | NA | NA | 17 | NA |
| 2 | Basu et al.^35^ | 45 | NA | 45 |  | NA | NA | NA | 33 | NA | NA | 33 | NA | NA | NA |
| 3 | Bauer et al.^5^ | 40 | 17 | 25 | 25 | NA | NA | NA | 29 | 19 | 13 | 15 | NA | NA | NA |
| 4 | Bode et al. 1984^7^ | 10 | NA | NA | NA | NA | NA | NA | 4 | NA | NA | NA | NA | NA | NA |
| 5 | Chesta et al. 1993^9^ | 16 | NA | NA | NA | NA | NA | NA | 7 | NA | NA | NA | NA | NA | NA |
| 6 | Chesta et al. 1991^10^ | 36 | NA | NA | NA | NA | NA | NA | 19 | NA | NA | NA | NA | NA | NA |
| 7 | Gunnarsdottir et al.^33^ | 24 | 16 | 8 | 12 | NA | NA | NA | 4 | 4 | 3 | 1 | NA | NA | NA |
| 8 | Jun et al.^14^ | 53 | 32 | 21 | 16 | 2 | 9 | NA | 32 | 13 | 17 | 15 | 1 | 8 | NA |
| 9 | Kiow et al.^16^ | 4 | NA | NA | 4 | NA | 1 | NA | 1 | 1 | NA | NA | NA | 0 | NA |
| 10 | Lakshmi et al.^17^ | 174 | 70 | 104 | 96 | NA | 19 | 64 | 42 | 28 | 13 | 29 | NA | 4 | 15 |
| 11 | Liu et al.^18^ | 150 | 30 | 60 | NA | 30 | NA | NA | 66 | NA | 6 | 36 | 13 | NA | NA |
| 12 | Morencos et al.^20^ | 89 | 23 | 66 | 70 | 12 | NA | NA | 27 | 26 | 3 | 24 | 8 | NA | NA |
| 13 | Pande et al.^21^ | 53 | 15 | 38 | 22 | 6 | NA | 20 | 26 | 16 | 3 | 23 | 3 | NA | 12 |
| 14 | Scarpellini et al. 2010^24^ | 56 | 18 | 34 | 22 | 10 | NA | NA | 29 | 14 | 6 | 21 | 9 | NA | NA |
| 15 | Scarpellini et al. 2022^25^ | 22 | 6 | 16 | 18 | NA | NA | 5 | 9 | NA | NA | NA | NA | NA | NA |
| 16 | Shindo et al.^27^ | 27 | NA | NA | 7 | NA | NA | NA | 9 | 3 | NA | NA | NA | NA | NA |
| 17 | Steed et al.^28^ | 23 | NA | NA | NA | NA | NA | NA | 0 | NA | NA | NA | NA | NA | NA |
| 18 | Xinpeng et al.^30^ | 47 | 11 | 36 | NA | NA | NA | NA | 22 | NA | 2 | 20 | NA | NA | NA |
| 19 | Yang et al.^31^ | 45 | NA | NA | NA | NA | NA | NA | 16 | NA | NA | NA | NA | NA | NA |
| 20 | Yao et al.^32^ | 120 | NA | NA | 20 | NA | NA | NA | 53 | 0 | NA | NA | NA | NA | NA |

CLD: chronic liver disease; SIBO: small intestinal bacterial overgrowth; PHT: portal hypertension; SBP: spontaneous bacterial peritonitis; HE: hepatic encephalopathy; VB: variceal bleeding; n: number; NA: not applicable.

**Table S3:** SIBO in patients with CLD, stratified by aetiology of liver disease.

| No | Author | MASLD n | MASH n | ALD  n | Viral hepatitis n | | Autoimmune liver disease n | | | Cryptogenic liver disease n | SIBO in MASLD n | SIBO in MASH n | SIBO in ALD n | SIBO in Viral hepatitis n | | SIBO in Autoimmune liver disease n | | | SIBO in Cryptogenic liver disease n |
| --- | --- | --- | --- | --- | --- | --- | --- | --- | --- | --- | --- | --- | --- | --- | --- | --- | --- | --- | --- |
|  |  |  |  |  | HCV | HBV | PSC | PBC | AIH |  |  |  |  | HCV | HBV | PSC | PBC | AIH |  |
| 1 | Bjornsson et al.^6^ | NA | NA | NA | NA | NA | 22 | NA | NA | NA | NA | NA | NA | NA | NA | 1 | NA | NA | NA |
| 2 | Bode et al.^8^ | NA | NA | 24 | NA | NA | NA | NA | NA | NA | NA | NA | 8 | NA | NA | NA | NA | NA | NA |
| 3 | Bode et al.^7^ | NA | NA | 27 | NA | NA | NA | NA | NA | NA | NA | NA | 13 | NA | NA | NA | NA | NA | NA |
| 4 | Chesta et al.^10^ | NA | NA | 23 | NA | NA | NA | NA | NA | 13 | NA | NA | 16 | NA | NA | NA | NA | NA | 5 |
| 5 | Fialho et al.^11^ | 104 | NA | NA | NA | NA | NA | NA | NA | NA | 64 | NA | NA | NA | NA | NA | NA | NA | NA |
| 6 | Fitriakusumah et al.^12^ | 115 | NA | NA | NA | NA | NA | NA | NA | NA | 36 | NA | NA | NA | NA | NA | NA | NA | NA |
| 7 | Ghoshal et al.^13^ | NA | 35 | NA | NA | NA | NA | NA | NA | NA | NA | 7 | NA | NA | NA | NA | NA | NA | NA |
| 8 | Gunnarsdottir et al.^33^ | NA | 2* | 10 | 12 | NA | 1 | NA | NA | 3 | NA | 1* | 2 | 2 | NA | 0 | NA | NA | 0 |
| 9 | Kapil et al.^15^ | 32 | 10 | NA | NA | NA | NA | NA | NA | NA | 12 | 3 | NA | NA | NA | NA | NA | NA | NA |
| 10 | Kiow et al.^16^ | NA | NA | NA | NA | NA | NA | 58 | NA | NA | NA | NA | NA | NA | NA | NA | 19 | NA | NA |
| 11 | Lakshmi et al.^17^ | NA | NA | 47 | 21 | 29 | NA | NA | 22 | 52 | NA | NA | 10 | 6 | 7 | NA | NA | 7 | 11 |
| 12 | Liu et al.^18^ | NA | NA | NA | NA | 150 | NA | NA | NA | NA | NA | NA | NA | NA | 66 | NA | NA | NA | NA |
| 13 | Mao et al.^36^ | 46 | NA | NA | NA | NA | NA | NA | NA | NA | 29 | NA | NA | NA | NA | NA | NA | NA | NA |
| 14 | Miele et al.^19^ | 35 | 17 | NA | NA | NA | NA | NA | NA | NA | 21 | 10 | NA | NA | NA | NA | NA | NA | NA |
| 15 | Morencos et al.^20^ | NA | NA | 89 | NA | NA | NA | NA | NA | NA | NA | NA | 27 | NA | NA | NA | NA | NA | NA |
| 16 | Nongthongbam et al.^37^ | 171 | NA | NA | NA | NA | NA | NA | NA | NA | 35 | NA | NA | NA | NA | NA | NA | NA | NA |
| 17 | Pande et al.^21^ | NA | NA | 13 | 8 | 18 | NA | NA | NA | 12 | NA | NA | 7 | 1 | 10 | NA | NA | NA | 7 |
| 18 | Sabate et al.^22^ | 127 | 31 | NA | NA | NA | NA | NA | NA | NA | 20 | 6 | NA | NA | NA | NA | NA | NA | NA |
| 19 | Sajjad et al.^23^ | NA | 12 | NA | NA | NA | NA | NA | NA | NA | NA | 6 | NA | NA | NA | NA | NA | NA | NA |
| 20 | Shanab et al.^38^ | NA | 18 | NA | NA | NA | NA | NA | NA | NA | NA | 14 | NA | NA | NA | NA | NA | NA | NA |
| 21 | Shi et al.^26^ | 103 | NA | NA | NA | NA | NA | NA | NA | NA | 60 | NA | NA | NA | NA | NA | NA | NA | NA |
| 22 | Steed et al.^28^ | NA | 2 | 14 | 1 | 1 | NA | 2 | 1 | NA | NA | 0 | 0 | 0 | 0 | NA | 0 | 0 | NA |
| 23 | Wigg et al.^29^ | NA | 22 | NA | NA | NA | NA | NA | NA | NA | NA | 11 | NA | NA | NA | NA | NA | NA | NA |
| 24 | Xinpeng et al.^30^ | NA | NA | NA | NA | 47 | NA | NA | NA | NA | NA | NA | NA | NA | 22 | NA | NA | NA | NA |
| 25 | Yang et al.^31^ | NA | NA | 6 | 14 | 22 | NA | NA | NA | 3 | NA | NA | 3 | 14 | 8 | NA | NA | NA | 1 |

CLD: chronic liver disease; SIBO: small intestinal bacterial overgrowth; MASLD: Metabolic dysfunction-associated steatotic liver disease; MASH: Metabolic dysfunction-associated steatohepatitis; ALD: alcoholic liver disease; HBV: hepatitis B virus; HCV: hepatitis C virus; PSC: primary sclerosing cholangitis; PBC: primary biliary cirrhosis; AIH: autoimmune hepatitis; n: number; MD: missing data; NA: not available. *MASH cirrhosis.

**Table S4:** Assessment of risk factors for SIBO in patients with CLD and controls in the studies included in this meta-analysis.

| **No** | **Author** | **Prior antibiotic use** | **Concurrent PPI use** | **Treatment with antibiotic** | **Duration of treatment** | **Treatment efficacy** | **Prior surgery** |
| --- | --- | --- | --- | --- | --- | --- | --- |
| 1 | Abid et al.^4^ | None within prior 4 weeks | NA | Rifaxamin 1200mg daily | 1 week | 94.1% symptomatic improvement. 100% normalization of breath tests | NA |
| 2 | Basu et al.^39^ | None | No | No | NA | NA | NA |
| 3 | Bauer et al.^5^ | None within prior 2 weeks | NA | No | NA | NA | NA |
| 4 | Bjornsson et al.^6^ | NA | NA | No | NA | NA | NA |
| 5 | Bode 1993 et al.^8^ | NA | NA | No | NA | NA | NA |
| 6 | Bode 1984 et al.^7^ | None within prior 4 weeks | No | No | NA | NA | NA |
| 7 | Chesta 1993 et al.^9^ | None within prior 4 weeks | Excluded if used antihistamine within prior 4 weeks | Tetracycline 2g daily | 2 weeks | No re-testing for efficacy | Excluded |
| 8 | Chesta 1991 et al.^40^ | None within prior 4 weeks | Excluded if used antihistamine within prior 4 weeks | No | NA | NA | NA |
| 9 | Fialho et al.^11^ | NA | NA | No | NA | NA | NA |
| 10 | Fitriakusumah et al.^12^ | None within prior 4 weeks | NA | No | NA | NA | Excluded |
| 11 | Ghoshal et al.^13^ | None within prior 4 weeks | NA | No | NA | NA | NA |
| 12 | Gunnarsdottir et al.^33^ | NA | NA | No | NA | NA | NA |
| 13 | Jun et al.^14^ | None within prior 4 weeks | No | No | NA | NA | Excluded |
| 14 | Kapil et al.^15^ | NA | NA | No | NA | NA | NA |
| 15 | Kiow et al.^16^ | None within prior 4 weeks | NA | No | NA | NA | Excluded |
| 16 | Lakshmi et al.^17^ | None within prior 4 weeks | NA | No | NA | NA | NA |
| 17 | Liu et al.^18^ | NA | NA | No | NA | NA | NA |
| 18 | Mao et al.^36^ | NA | NA | No | NA | NA | NA |
| 19 | Miele et al.^19^ | None within prior 8 weeks | No | No | NA | NA | Excluded |
| 20 | Morencos et al.^20^ | None within prior 4 weeks | NA | No | NA | NA | Excluded |
| 21 | Nongthongbam et al.^37^ | NA | NA | No | NA | NA | NA |
| 22 | Pande et al.^21^ | None within prior 1 week | No | No | NA | NA | NA |
| 23 | Sabate et al.^22^ | None within prior 4 weeks | NA | No | NA | NA | Excluded |
| 24 | Sajjad et al.^23^ | NA | NA | Ciprofloxacin 500mg twice daily | 5 days | 5/6 (83.3%) normalization of breath tests | NA |
| 25 | Scarpellini 2010 et al.^41^ | None within prior 2 weeks | NA | No | NA | NA | Excluded |
| 26 | Scarpellini 2022 et al.^25^ | None within prior 4 weeks | No | No | NA | NA | Excluded |
| 27 | Shanab et al.^38^ | None within prior 4 weeks | No | No | NA | NA | Excluded |
| 28 | Shi et al.^26^ | No | NA | No | NA | NA | Excluded |
| 29 | Shindo et al.^27^ | NA | NA | No | NA | NA | NA |
| 30 | Steed et al.^28^ | None within prior 12 weeks | NA | No | NA | NA | NA |
| 31 | Wigg et al.^29^ | None within prior 4 weeks | NA | No | NA | NA | NA |
| 32 | Xinpeng et al.^30^ | NA | NA | No | NA | NA | Excluded |
| 33 | Yang et al.^31^ | None within prior 4 weeks | No | No | NA | NA | Excluded |
| 34 | Yao et al.^32^ | None within prior 6 weeks | NA | No | NA | NA | NA |

CLD: chronic liver disease; SIBO: small intestinal bacterial overgrowth; GBT: glucose breath test; LBT: lactulose breath test; NA: not applicable.

**Table S5:** Assessment of cut off criteria for diagnosing SIBO in patients with CLD and controls.

| **Study No** | **Author** | **Mode of diagnosis of SIBO** | **Dose of substrate** | **Cut off criteria for SIBO diagnosis** |
| --- | --- | --- | --- | --- |
| 1 | Abid et al.^4^ | LBT | 50g | Increase ≥20ppm H_2_ rise from baseline |
| 2 | Basu et al.^39^ | LBT | NA | NA |
| 3 | Bauer et al.^5^ | GBT | 75g | **Criteria 1:** Fasting breath H_2_ >20ppm or increase in breath H_2_ by >20ppm.  **Criteria 2:** Fasting breath H_2_ >20ppm or increase in breath H_2_ by >12ppm.  **Criteria 3:** Increase in breath H_2_ by >13ppm |
| 3 | Bauer et al.^5^ | Jejunal Culture |  | Total upper jejunal colony count ≥10^5^ cfu/ml or colonic flora count ≥10^3^cfu/ml |
| 4 | Bjornsson et al.^6^ | Jejunal Culture |  | Total colonic flora count >10^5^ cfu/ml |
| 5 | Bode 1993 et al.^8^ | LBT | 10g | NA |
| 6 | Bode 1984 et al.^7^ | Jejunal Culture |  | Total bacterial count >10^5^ cfu/ml |
| 7 | Chesta 1993 et al.^9^ | LBT | 12g | Increase in early peak H_2_ >10ppm in two consecutive measurements in the frst 60 minutes distinguishable from colonic peak. |
| 8 | Chesta 1991 et al.^40^ | LBT | 15g | **Criteria 1:** Elevated basal H_2_ >2 SD above the control group (>16ppm)  **Criteria 2:** Early H_2_ rise >10ppm compared to baseline in two consecutive measurements during the test  **Criteria 3:** Cumulative expired H_2_ greater than the mean + 2SD of the control group in the first 40 minutes. |
| 8 | Chesta 1991 et al.^40^ | Jejunal Culture |  | Total aerobic bacterial count >10^6^ cfu/ml or total anaerobic bacterial count >10^5^ cfu/ml. |
| 9 | Fialho et al.^11^ | GBT | 50g | H_2_/CH4 increase >20ppm above baseline or H_2_/CH4 increase >12ppm between minimal and maximal values. |
| 10 | Fitriakusumah et al.^12^ | NA |  | Increase in H_2_ >20ppm from baseline |
| 11 | Ghoshal et al.^13^ | GBT | 100g | Increase in H_2_ >12ppm above basal levels in two consecutive readings |
| 11 | Ghoshal et al.^13^ | Jejunal Culture |  | **High-grade SIBO:** Total bacterial count >10^5^ cfu/ml  **Low-grade SIBO:** Total bacterial count >10^3^ cfu/ml but <10^5^ cfu/ml |
| 12 | Gunnarsdottir et al.^33^ | Jejunal Culture |  | Total colonic flora count >10^5^ cfu/ml |
| 13 | Jun et al.^14^ | LBT | 15g | Basal H_2_ value of >20 ppm or early H_2_ peak of >20 ppm (>10 ppm in the case of CH4) in the first 90 min after lactulose ingestion followed by a second large peak or plateau |
| 14 | Kapil et al.^15^ | DU Culture |  | Total bacterial count >10^5^ cfu/ml |
| 15 | Kiow et al.^16^ | LBT | 10g | **Criteria 1:** Increase H_2_/CH4 >20ppm from baseline  **Criteria 2:** >13ppm on two consecutive measures earlier than 90mins  **Criteria 3:** Presence of two increases (>20ppm from baseline or two consecutive increases >13ppm) one of which occurs earlier than 90mins |
| 16 | Lakshmi et al.^17^ | GBT | 100g | Increase in H_2_ >12ppm above basal levels in two consecutive readings |
| 17 | Liu et al.^18^ | LBT | 10g | **Criteria 1:** Baseline H_2_ >20ppm  **Criteria 2:** H_2_ >32ppm at any point during the test. |
| 18 | Mao et al.^36^ | LBT | NA | NA |
| 19 | Miele et al.^19^ | GBT | 50g | Increase in H_2_ >12ppm above basal levels |
| 20 | Morencos et al.^20^ | GBT | 50g | Increase in H_2_ >20ppm in basal conditions or increase >20ppm from baseline |
| 21 | Nongthongbam et al.^37^ | NA | NA | Basal H_2_ >20ppm or an increase in H_2_ >12ppm from baseline |
| 22 | Pande et al.^21^ | GBT | 100g | Basal H_2_ >20ppm or increase >12ppm from baseline |
| 23 | Sabate et al.^22^ | GBT | 50g | Fasting H_2_ concentrations >20ppm or increase in H_2_ >12ppm above baseline in the first 2 hours |
| 24 | Sajjad et al.^23^ | GBT | 75g | Increase in H_2_ >20ppm above baseline |
| 25 | Scarpellini 2010 et al.^41^ | GBT | NA | Early rise in H_2_ >20ppm in the first 90 minutes |
| 26 | Scarpellini 2022 et al.^25^ | LBT | 10g | Early rise in H_2_ >20ppm in the first 90 minutes of the test. |
| 27 | Shanab et al.^38^ | LBT | 10g | **Criteria 1:** Baseline H_2_/CH4 >20ppm  **Criteria 2:** Increase in H_2_/CH4 >20ppm from baseline |
| 28 | Shi et al.^26^ | LBT | 10g | **Criteria 1:** Baseline H_2_ >20ppm and remains >20ppm after 30 minutes.  **Criteria 2:** Peak above baseline >12ppm within 60 minutes with a second peak >12ppm after 60 minutes  **Criteria 3:** Increase >12ppm above baseline within 60 minutes |
| 29 | Shindo et al.^27^ | Jejunal Culture |  | Total bacterial count >10^5^ cfu/ml |
| 30 | Steed et al.^28^ | DU Biopsy and RT-PCR |  | NA |
| 31 | Wigg et al.^29^ | C-D-Xylose/LBT | 1μCi C-D-xylose + 6.68g lactulose | **Criteria 1**: >70 x 10^-6^ DPM of CO_2_ was expired before the colonic H_2_ and CH4 rise.  **Criteria 2**: A double H_2_/CH4 peak occurred. |
| 32 | Xinpeng et al.^30^ | LBT | 10g | **Criteria 1:** Baseline H_2_ >20ppm  **Criteria 2:** Rise in H_2_ >12ppm from baseline |
| 33 | Yang et al.^31^ | GBT | 50g | Increase in H_2_ >13ppm or increase in CH4 >12ppm |
| 34 | Yao et al.^32^ | GBT | 50g | Increase in H_2_ >12ppm or fasting H_2_ ≥20ppm |

ppm: parts per million; LBT: lactulose breath test; GBT: glucose breath test; DU: duodenum; H_2_: hydrogen; CH_4_: methane; cfu/ml: colony forming unit/ml, NA: not applicable.

**Table S6:** Studies showing the prevalence of SIBO in patients with CLD with cirrhosis, stratified according to Child-Turcotte-Pugh (CTP) score.

| No | Author | CLD patients with cirrhosis, n | CLD patients with cirrhosis, n | | | SIBO in CLD patients with cirrhosis, n | SIBO in CLD patients with cirrhosis, n | | |
| --- | --- | --- | --- | --- | --- | --- | --- | --- | --- |
|  |  |  | CP-A | CP-B | CP-C |  | CP-A | CP-B | CP-C |
| 1 | Bauer et al.^5^ | 40 | 17 | 14 | 9 | 29 | 13 | 9 | 7 |
| 2 | Bode 1984 et al.^7^ | 10 | 10 | NA | NA | 4 | 4 | NA | NA |
| 3 | Gunnarsdottir et al.^33^ | 24 | 16 | 8 | NA | 4 | 3 | 1 | NA |
| 4 | Jun et al.^14^ | 53 | 32 | 13 | 8 | 32 | 17 | 9 | 6 |
| 5 | Lakshmi et al.^17^ | 174 | 70 | 67 | 37 | 42 | 13 | 16 | 13 |
| 6 | Liu et al.^18^ | 150 | 30 | 30 | 30 | 66 | 6 | 14 | 22 |
| 7 | Morencos et al.^20^ | 89 | 23 | 37 | 29 | 27 | 3 | 10 | 14 |
| 8 | Pande et al.^21^ | 53 | 15 | 23 | 15 | 26 | 3 | 12 | 11 |
| 9 | Scarpellini 2022 et al.^25^ | 22 | 18 | 19 | 15 | 9 | 6 | 11 | 10 |
| 10 | Xinpeng et al.^30^ | 47 | 11 | 16 | 20 | 22 | 2 | 7 | 13 |

SIBO: small intestinal bacterial overgrowth; CLD: chronic liver disease; NA: not available; n: number.

**Table S7:** Studies assessing the effect of SIBO on synthetic functions in patients with CLD.

| **No** | **Author** | **CLD patients with SIBO, n** | **CLD without SIBO, n** | **Serum albumin (g/L) in CLD patients with SIBO, mean (SD)** | **Serum albumin (g/L) in CLD patients without SIBO, mean (SD)** | **Serum bilirubin (umol/L) in CLD patients with SIBO, mean (SD)** | **Serum bilirubin (umol/L) in CLD patients without SIBO, mean (SD)** | **Coagulation profile* in CLD patients with SIBO, mean (SD)** | **Coagulation profile* in CLD patients without SIBO, mean (SD)** |
| --- | --- | --- | --- | --- | --- | --- | --- | --- | --- |
| 1 | Abid et al.^4^ | 55 | 35 | 27 (3.2) | 28 (3.9) | 44.5(11.3) | 42.75 (13.3) | 15.4 (3.0) | 15.4 (2.5) |
| 2 | Bjornnson et al.^6^ | 1 | 23 | 43 (NA) | 40.52 (3.9) | 6 (NA) | 25.3 (39.8) | 1 (NA) | 0.97 (0.2) |
| 3 | Fialho et al.^11^ | 141 | 231 | 41.2 (0.4) | 45.6 (2.1) | NA | NA | NA | NA |
| 4 | Ghoshal et al.^13^ | 30 | 5 | 44.5 (3.5) | 40 (6.1) | NA | NA | NA | NA |
| 5 | Lakshmi et al.^17^ | 42 | 132 | 34 (8) | 34 (6) | 41.0 (51.3) | 42.75 (65.0) | 18.9 (4.8) | 17.6 (3.0) |
| 7 | Wigg et al.^29^ | 22 | 23 | NA | NA | 18 (23) | 11(5) | NA | NA |
| 8 | Yang et al.^31^ | 16 | 29 | 28 (7) | 31 (6) | 53.01 (49.6) | 70.11 (34.2) | 14.9 (1.4) | 14.1 (2.0) |

SIBO: small intestinal bacterial overgrowth; CLD: chronic liver disease; SD: standard deviation; NA: not applicable, n: number, Coagulation profile was expressed as prothrombin time.

**Table S6:** Newcastle-Ottawa scale for assessment of quality of Case control studies included in the Systematic review and meta-analysis.

| **SELECTION** | **1^4^** | **2^39^** | **3^5^** | **4^6^** | **5^7^** | **6^8^** | **7^40^** | **8^9^** | **9^11^** | **10^12^** | **11^13^** | **12^33^** | **13^14^** | **14^15^** | **15^16^** | **16^17^** | **17^18^** |
| --- | --- | --- | --- | --- | --- | --- | --- | --- | --- | --- | --- | --- | --- | --- | --- | --- | --- |
| Is the case definition adequate? | 1 | 1 | 1 | 1 | 1 | 1 | 1 | 1 | 1 | 1 | 1 | 1 | 1 | 1 | 1 | 1 | 1 |
| Representativeness of the cases | 1 | 1 | 1 | 1 | 1 | 1 | 1 | 1 | 1 | 1 | 1 | 1 | 1 | 1 | 1 | 1 | 1 |
| Selection of Controls | 1 | 0 | 1 | 1 | 0 | 1 | 1 | 1 | 0 | 0 | 1 | 0 | 1 | 1 | 1 | 1 | 1 |
| Definition of Controls | 1 | 0 | 1 | 1 | 1 | 1 | 1 | 1 | 1 | 0 | 1 | 0 | 1 | 1 | 1 | 1 | 1 |
| **COMPARIBILITY** |  |  |  |  |  |  |  |  |  |  |  |  |  |  |  |  |  |
| Study controls for single factor | 1 | 0 | 1 | 1 | 1 | 0 | 0 | 1 | 1 | 1 | 1 | 1 | 1 | 1 | 1 | 1 | 1 |
| Study controls for additional factors | 1 | 0 | 1 | 1 | 0 | 0 | 0 | 1 | 1 | 1 | 1 | 0 | 1 | 1 | 1 | 1 | 1 |
| **EXPOSURE** | 1 | 1 | 1 | 1 | 1 | 1 | 1 | 1 | 1 | 1 | 1 | 1 | 1 | 1 | 1 | 1 | 1 |
| Ascertainment of exposure (presence of SIBO) | 1 | 1 | 1 | 1 | 1 | 1 | 1 | 1 | 1 | 1 | 1 | 1 | 1 | 1 | 1 | 1 | 1 |
| Same method of ascertainment for cases and controls | 1 | 0 | 1 | 1 | 0 | 1 | 1 | 1 | 0 | 0 | 1 | 0 | 1 | 1 | 1 | 1 | 1 |
| Non-Response rate | 1 | 0 | 1 | 1 | 1 | 1 | 1 | 1 | 1 | 0 | 1 | 0 | 1 | 1 | 1 | 1 | 1 |
| **Overall Quality Score (Maximum = 9)** | 9 | 4 | 9 | 9 | 7 | 6 | 6 | 9 | 8 | 7 | 9 | 5 | 9 | 9 | 8 | 9 | 8 |

| **SELECTION** | **18^36^** | **19^19^** | **20^20^** | **21^37^** | **22^21^** | **23^22^** | **24^23^** | **25^41^** | **26^25^** | **27^38^** | **28^26^** | **29^27^** | **30^28^** | **31^29^** | **32^30^** | **33^31^** | **34^32^** |
| --- | --- | --- | --- | --- | --- | --- | --- | --- | --- | --- | --- | --- | --- | --- | --- | --- | --- |
| Is the case definition adequate? | 1 | 1 | 1 | 1 | 1 | 1 | 1 | 1 | 1 | 1 | 1 | 1 | 1 | 1 | 1 | 1 | 1 |
| Representativeness of the cases | 1 | 1 | 1 | 1 | 1 | 1 | 1 | 1 | 1 | 1 | 1 | 1 | 1 | 1 | 1 | 1 | 1 |
| Selection of Controls | 1 | 1 | 1 | 0 | 1 | 1 | 1 | 1 | 1 | 0 | 1 | 1 | 1 | 1 | 1 | 1 | 1 |
| Definition of Controls | 0 | 1 | 1 | 0 | 1 | 1 | 1 | 1 | 0 | 1 | 0 | 1 | 1 | 1 | 0 | 1 | 1 |
| **COMPARIBILITY** |  |  |  |  |  |  |  |  |  |  |  |  |  |  |  |  |  |
| Study controls for single factor | 0 | 1 | 1 | 0 | 1 | 1 | 1 | 1 | 1 | 0 | 1 | 0 | 1 | 1 | 1 | 1 | 1 |
| Study controls for additional factors | 0 | 1 | 1 | 0 | 1 | 1 | 1 | 1 | 0 | 0 | 1 | 0 | 1 | 1 | 1 | 1 | 0 |
| **EXPOSURE** |  |  |  |  |  |  |  |  |  |  |  |  |  |  |  |  |  |
| Ascertainment of exposure (presence of SIBO) | 1 | 1 | 1 | 1 | 1 | 1 | 1 | 1 | 1 | 1 | 1 | 1 | 1 | 1 | 1 | 1 | 1 |
| Same method of ascertainment for cases and controls | 1 | 1 | 1 | 1 | 1 | 1 | 1 | 1 | 1 | 1 | 1 | 1 | 1 | 1 | 1 | 1 | 1 |
| Non-Response rate | 0 | 1 | 1 | 0 | 1 | 1 | 1 | 1 | 1 | 0 | 0 | 0 | 1 | 1 | 0 | 1 | 0 |
| **Overall Quality Score (Maximum = 9)** | 5 | 9 | 9 | 4 | 9 | 9 | 9 | 9 | 7 | 5 | 7 | 6 | 9 | 9 | 7 | 9 | 7 |

**Table S9:** Studies showing the prevalence of intestinal methanogen overgrowth in patients with CLD and controls.

| **Study No** | **Author** | **Mode of diagnosis of SIBO** | **Patients with CLD, n** | **Controls, n** | **Methane positive in cases, n** | **Methane positive in controls, n** |
| --- | --- | --- | --- | --- | --- | --- |
| 1 | Jun et al.**^14^** | LBT | 53 | 42 | 4 | 2 |
| 2 | Shanab et al.^38^ | LBT | 18 | 16 | 2 | 2 |
| 3 | Yang et al.^31^ | GBT | 45 | 28 | 6 | 0 |

CLD: chronic liver disease; LBT: lactulose breath test; GBT: glucose breath test; n: number; NA: not applicable.

**Table S10:** Studies assessing the effect of PPI on SIBO prevalence in patients with CLD.

| **No** | **Author** | **CLD, n** | **SIBO in CLD, n** | **Mode of diagnosis of SIBO** | **CLD patients on PPI, n** | **SIBO in CLD patients on PPI, n** | **CLD patients not on PPI, n** | **SIBO in CLD patients not on PPI, n** |
| --- | --- | --- | --- | --- | --- | --- | --- | --- |
| 1 | Bauer et al.^5^ | 40 | 29 | Jejunal aspirate + HBT | 17 | 17 | 23 | 2 |
| 2 | Gunnarsdottir et al.^33^ | 24 | 4 | Jejunal aspirate | 1 | 0 | 23 | 4 |
| 3 | Lakshmi et al.^17^ | 174 | 42 | GHBT | 62 | 16 | 112 | 26 |

CLD: chronic liver disease; PPI: proton pump inhibitor; SIBO: small intestinal bacterial overgrowth; NA: not applicable, n: number; GHBT: glucose hydrogen breath test.

**Table S11:** Studies evaluating the effect of antibiotic treatment in patients with CLD with SIBO.

| **Study No** | **Author** | **Patients who underwent antibiotic treatment, n** | **Treatment with antibiotic** | **Duration of treatment** | **Post treatment symptom improvement, n** | **Post treatment normalization of BT, n** |
| --- | --- | --- | --- | --- | --- | --- |
| 1 | Abid et al.^4^ | 28 | Rifaximin 1200mg daily | 7 days | 16/17* | 28 |
| 2 | Chest et al. 1993^9^ | 7 | Tetracycline 2g daily | 14 days | NA | NA |
| 3 | Sajjad et al.^23^ | 6 | Ciprofloxacin 500mg BD | 5 days | NA | 5 |

CLD: chronic liver disease; SIBO: small intestinal bacterial overgrowth; NA: not available; n: number; BD: two times a day; BT: breath test. *16 out of 17 patients had symptom assessment post treatment, were noted to have improved their symptoms.

**Table S12:** Studies assessing the effect of SIBO on intestinal permeability in patients with CLD.

| **No** | **Author** | **Method of intestinal permeability evaluation** | **CLD patients with SIBO, n** | **CLD patients with increased intestinal permeability and SIBO, % (n)** |
| --- | --- | --- | --- | --- |
| 1 | Miele et al.^19^ | Chromium-51 EDTA urinary excretion | 21 | 88.8% (16/18) |
| 2 | Scarpellini 2010 et al.^41^ | Chromium-51 EDTA urinary excretion | 10 | 100% (10/10) |
| 3 | Wigg et al.^29^ | Lactulose/ rhamnose ratio | 11 | NA |
| 4 | Bjornnson et al.^6^ | Lactulose/ rhamnose ratio | 1 | 0% (0/1) |

SIBO: small intestinal bacterial overgrowth; CLD: chronic liver disease; Cr51: Chromium-51; EDTA: Ethylenediaminetetraacetic acid; NA: not available; n: number.

**Table S13:** Studies assessing the effect of SIBO on oro-cecal transit time in patients with CLD.

| **No** | **Author** | **CLD patients, n** | **Controls, n** | **CLD patients with SIBO, n** | **CLD patients without SIBO, n** | **OCTT in CLD patients, mean (SD)** | **OCTT in controls, mean (SD)** | **OCTT in CLD patients with SIBO, mean (SD)** | **OCTT in CLD patients without SIBO, mean (SD)** |
| --- | --- | --- | --- | --- | --- | --- | --- | --- | --- |
| 1 | Shi et al.^26^ | 103 | 49 | 60 | 43 | 142.18 (32.4) | 107.55 (22.9) | 154.1 (30.2) | 125.6 (27.8) |
| 2 | Wigg et al.^29^ | 22 | 23 | 11 | 12 | 182 (43) | 180 (53) | 196 (42.3) | 157.1 (52.5) |

CLD: chronic liver disease; SD: standard deviation, n: number; SIBO: small intestinal bacterial overgrowth.

**REFERENCES:**

1. Ghoshal UC, Sachdeva S, Ghoshal U, et al. Asian-Pacific consensus on small intestinal bacterial overgrowth in gastrointestinal disorders: An initiative of the Indian Neurogastroenterology and Motility Association. Indian J Gastroenterol 2022:1-25.

2. Pimentel M, Saad RJ, Long MD, et al. ACG Clinical Guideline: Small Intestinal Bacterial Overgrowth. Am J Gastroenterol 2020;115:165-178.

3. DerSimonian R, Laird N. Meta-analysis in clinical trials. Control Clin Trials 1986;7:177-88.

4. Abid S, Kamran M, Abid A, et al. Minimal Hepatic Encephalopathy: Effect of H. pylori infection and small intestinal bacterial overgrowth treatment on clinical outcomes. SCIENTIFIC REPORTS 2020;10:10079.

5. Bauer TM, Schwacha H, Steinbrückner B, et al. Diagnosis of small intestinal bacterial overgrowth in patients with cirrhosis of the liver: poor performance of the glucose breath hydrogen test. Journal of Hepatology 2000;33:382-386.

6. Björnsson E, Cederborg A, Åkvist A, et al. Intestinal permeability and bacterial growth of the small bowel in patients with primary sclerosing cholangitis. Scandinavian Journal of Gastroenterology 2005;40:1090-1094.

7. Bode JC, Bode C, Heidelbach R, et al. Jejunal microflora in patients with chronic alcohol abuse. Hepatogastroenterology 1984;31:30-4.

8. Bode C, Kolepke R, Schafer K, et al. Breath hydrogen excretion in patients with alcoholic liver disease--evidence of small intestinal bacterial overgrowth. Z Gastroenterol 1993;31:3-7.

9. Chesta J, Defilippi C, Defilippi C. Abnormalities in proximal small bowel motility in patients with cirrhosis. Hepatology 1993;17:828-832.

10. Chesta J, Silva M, Thompson L, et al. [Bacterial overgrowth in small intestine in patients with liver cirrhosis]. Revista medica de Chile 1991;119:626-632.

11. Fialho A, Fialho A, Thota P, et al. Small Intestinal Bacterial Overgrowth Is Associated with Non-Alcoholic Fatty Liver Disease. JOURNAL OF GASTROINTESTINAL AND LIVER DISEASES 2016;25:159-165.

12. Yoga F, Lesmana CRA, Winda Permata B, et al. The role of Small Intestinal Bacterial Overgrowth (SIBO) in Non-alcoholic Fatty Liver Disease (NAFLD) patients evaluated using Controlled Attenuation Parameter (CAP) Transient Elastography (TE): a tertiary referral center experience. BMC Gastroenterology 2019;19:1-7.

13. Ghoshal U, Baba C, Ghoshal U, et al. Low-grade small intestinal bacterial overgrowth is common in patients with non-alcoholic steatohepatitis on quantitative jejunal aspirate culture. Indian Journal of Gastroenterology 2017;36:390-399.

14. Dae Won J, Kyung Tae K, Oh Young L, et al. Association between small intestinal bacterial overgrowth and peripheral bacterial DNA in cirrhotic patients. Digestive Diseases & Sciences 2010;55:1465-1471.

15. Kapil S, Duseja A, Sharma B, et al. Small Intestinal Bacterial Overgrowth and Toll Like Receptor Signaling in Patients with Nonalcoholic Fatty Liver Disease. Journal of Clinical and Experimental Hepatology 2015;5:S25-S25.

16. Kiow JLC, Vincent C, Sidani S, et al. High occurrence of small intestinal bacterial overgrowth in primary biliary cholangitis. NEUROGASTROENTEROLOGY AND MOTILITY 2019:e13691.

17. Lakshmi CP, Ghoshal UC, Kumar S, et al. Frequency and factors associated with small intestinal bacterial overgrowth in patients with cirrhosis of the liver and extra hepatic portal venous obstruction. Digestive Diseases & Sciences 2010;55:1142-1148.

18. Liu HS, Z; Zhang, Y. L; Zhang, H. Correlation between small intestinal bacterial growth and spontaneous peritonitis, liver function and disease severity in patients with post-hepatitis B cirrhosis. Journal of Biological Regulators & Homestatic Agents 2021;35:7.

19. Miele L, Valenza V, La Torre G, et al. Increased intestinal permeability and tight junction alterations in nonalcoholic fatty liver disease. Hepatology (Baltimore, Md.) 2009;49:1877-1887.

20. Casafont Morencos F, de las Heras Castano G, Martín Ramos L, et al. Small bowel bacterial overgrowth in patients with alcoholic cirrhosis. Digestive Diseases and Sciences 1995;40:1252-1256.

21. Pande C, Kumar A, Sarin SK. Small-intestinal bacterial overgrowth in cirrhosis is related to the severity of liver disease. Alimentary pharmacology & therapeutics 2009;29:1273-1281.

22. Sabaté J-M, Jouët P, Harnois F, et al. High prevalence of small intestinal bacterial overgrowth in patients with morbid obesity: a contributor to severe hepatic steatosis. Obesity surgery 2008;18:371-377.

23. Sajjad A, Mottershead M, Syn WK, et al. Ciprofloxacin suppresses bacterial overgrowth, increases fasting insulin but does not correct low acylated ghrelin concentration in non-alcoholic steatohepatitis. Aliment Pharmacol Ther 2005;22:291-9.

24. Scarpellini E, Cazzato A, Gabrielli M, et al. T.N.12 RELATIONSHIP BETWEEN SMALL INTESTINAL BACTERIAL OVERGROWTH AND INTESTINAL PERMEABILITY IN CIRRHOSIS. Digestive and Liver Disease 2010;42:S19-S19.

25. Scarpellini E, Abenavoli L, Cassano V, et al. The Apparent Asymmetrical Relationship Between Small Bowel Bacterial Overgrowth, Endotoxemia, and Liver Steatosis and Fibrosis in Cirrhotic and Non-Cirrhotic Patients: A Single-Center Pilot Study. Frontiers in medicine 2022;9:872428.

26. Shi H, Mao L, Wang L, et al. Small intestinal bacterial overgrowth and orocecal transit time in patients of nonalcoholic fatty liver disease. EUROPEAN JOURNAL OF GASTROENTEROLOGY & HEPATOLOGY 2021;33:E535-E539.

27. Shindo K, Machida M, Miyakawa K, et al. A syndrome of cirrhosis, achlorhydria, small intestinal bacterial overgrowth, and fat malabsorption. The American journal of gastroenterology 1993;88:2084-2091.

28. Steed H, Macfarlane GT, Blackett KL, et al. Bacterial translocation in cirrhosis is not caused by an abnormal small bowel gut microbiota. FEMS Immunol Med Microbiol 2011;63:346-54.

29. Wigg AJ, Roberts-Thomson IC, Dymock RB, et al. The role of small intestinal bacterial overgrowth, intestinal permeability, endotoxaemia, and tumour necrosis factor alpha in the pathogenesis of non-alcoholic steatohepatitis. Gut 2001;48:206-11.

30. Xinpeng WEI, Xiao GAO, Yingjie MA. Relationship between small intestinal bacterial overgrowth and severity of with hepatitis B cirrhosis. Linchuang Gandanbing Zazhi 2016;32:716-719.

31. Yang CY, Chang CS, Chen GH. Small-intestinal bacterial overgrowth in patients with liver cirrhosis, diagnosed with glucose H2 or CH4 breath tests. Scand J Gastroenterol 1998;33:867-71.

32. Jia Y, Le C, Lili Y, et al. Nutrition status and small intestinal bacterial overgrowth in patients with virus-related cirrhosis. Asia Pacific Journal of Clinical Nutrition 2016;25:283-291.

33. Gunnarsdottir SA, Sadik R, Shev S, et al. Small intestinal motility disturbances and bacterial overgrowth in patients with liver cirrhosis and portal hypertension. The American Journal of Gastroenterology 2003;98:1362-1370.

34. Rezaie A, Buresi M, Lembo A, et al. Hydrogen and Methane-Based Breath Testing in Gastrointestinal Disorders: The North American Consensus. Am J Gastroenterol 2017;112:775-784.

35. Basu P, Mittimani K, Shah NJ, et al. Prevalence of Small Bowel Bacterial Over Growth (SIBO) in Decompensated Cirrhosis With Portal Hypertension: An Clinical Pilot Study. GASTROENTEROLOGY 2013;144:S1001-S1001.

36. Mao L, Zhu S, Wang L, et al. THE ROLE OF SMALL INTESTINAL BACTERIA OVERGROWTH IN NONALCOHOLIC FATTY LIVER DISEASE. GASTROENTEROLOGY 2019;156:S926-S926.

37. Nongthombam S, Nayak B, Kumar A, et al. Prevalence of Small Intestinal Bacterial Overgrowth (SIBO) and Insulin Resistance in Both Obese and Non Obese Non-Alcoholic Fatty Liver Disease (NAFLD) Patients. Journal of Clinical and Experimental Hepatology 2015;5:S23-S24.

38. Shanab A, Scully P, Crosbie O, et al. Small intestinal bacterial overgrowth in nonalcoholic steatohepatitis: association with toll-like receptor 4 expression and plasma levels of interleukin 8. Digestive Diseases & Sciences 2011;56:1524-1534.

39. Basu P, Mittimanj K, Shah NJ, et al. Prevalence of small bowel bacterial over growth (SIBO) in decompensated cirrhosis with portal hypertension: A clinical pilot study. Journal of Clinical and Experimental Hepatology 2013;3:S83-S84.

40. Chesta J, Silva M, Thompson L, et al. SMALL-INTESTINE BACTERIAL OVERGROWTH IN PATIENTS WITH HEPATIC CIRRHOSIS. REVISTA MEDICA DE CHILE 1991;119:626-632.

41. Scarpellini E, Cazzato A, Gabrielli M, et al. Relationship between Small Intestinal Bacterial Overgrowth and intestinal permeability in cirrhosis. HELICOBACTER 2010;15:339-340.
